# Reevaluating response and failure of medical treatment of endometriosis: a systematic review

**DOI:** 10.1016/j.fertnstert.2017.05.004

**Published:** 2017-07

**Authors:** Christian M. Becker, William T. Gattrell, Kerstin Gude, Sukhbir S. Singh

**Affiliations:** a Endometriosis Care Centre, Nuffield Department of Obstetrics and Gynaecology, University of Oxford, Oxford, United Kingdom; b Research Evaluation Unit, Oxford Pharmagenesis, Oxford, United Kingdom; c Department of Mechanical Engineering and Mathematical Sciences, Oxford Brookes University, Oxford, United Kingdom; d Medical Affairs Women's Healthcare, Bayer, Berlin, Germany; e Department of Obstetrics and Gynaecology, Ottawa Hospital Research Institute, Ottawa, Ontario, Canada

**Keywords:** Endometriosis, pain, medical therapy, systematic review

## Abstract

**Objective:**

To assess patient response rates to medical therapies used to treat endometriosis-associated pain.

**Design:**

A systematic review with the use of Medline and Embase.

**Setting:**

Not applicable.

**Patient(s):**

Women receiving medical therapy to treat endometriosis.

**Interventions(s):**

None.

**Main Outcome Measure(s):**

The proportions of patients who: experienced no reduction in endometriosis-associated pain symptoms; had pain symptoms remaining at the end of the treatment period; had pain recurrence after treatment cessation; experienced an increase or no change in disease score during the study; were satisfied with treatment; and discontinued therapy owing to adverse events or lack of efficacy. The change in pain symptom severity experienced during and after treatment, as measured on the visual analog scale, was also assessed.

**Result(s):**

In total, 58 articles describing 125 treatment arms met the inclusion criteria. Data for the response of endometriosis-associated pain symptoms to treatment were presented in only 29 articles. The median proportions of women with no reduction in pain were 11%–19%; at the end of treatment, 5%–59% had pain remaining; and after follow-up, 17%–34% had experienced recurrence of pain symptoms after treatment cessation. After median study durations of 2–24 months, the median discontinuation rates due to adverse events or lack of efficacy were 5%–16%.

**Conclusion(s):**

Few studies of medical therapies for endometriosis report outcomes that are relevant to patients, and many women gain only limited or intermittent benefit from treatment.

**Discuss:** You can discuss this article with its authors and with other ASRM members at **https://www.fertstertdialog.com/users/16110-fertility-and-sterility/posts/16307-23631**

Endometriosis is a chronic inflammatory disease that mainly affects women of reproductive age. Medical therapy can alleviate endometriosis-associated pain, but for many women pain relief is limited in efficacy and duration, and symptoms often reoccur after treatment cessation (1). Most current medical therapies for endometriosis to treat the disease and its symptoms rely on suppression of local or systemic estrogen levels or direct hormonal effects on endometriosis lesions. To date, all available hormonal therapies appear to have similar efficacy, but their tolerability profiles differ (2). The most widely used long-term therapies are progestins and combined oral contraceptives (COCs), but they are associated with irregular bleeding patterns, breast tenderness, and mood disturbances in some women (3). Other hormonal therapies include GnRH agonists, which induce a hypoestrogenic state resulting in menopausal symptoms, such as hot flushes, and are associated with reduced bone mineral density (4). They are therefore normally restricted to short-term use. Combination with add-back therapy may extend the period for which GnRH agonists can be used, although long-term safety data on this treatment regimen are limited (3). Danazol, especially when administered orally, is associated with significant androgenic side effects, which has considerably restricted its routine use. In addition, analgesics, such as nonsteroidal antiinflammatory drugs (NSAIDs), are widely prescribed for pain relief despite limited evidence of their efficacy in endometriosis (1).

It has been suggested that one-fourth to one-third of patients treated with the use of COCs or progestins require further treatment because of lack of response or poor tolerability 5, 6, but there are limited data to support these figures. A review of randomized controlled trials (RCTs) published in the period 1976–1998 showed that 40%–70% of women receiving surgical treatment or medical therapy had relief from endometriosis-associated pelvic pain for ≥6 months (7). In contrast, in a systematic review of the use of progestins published in 1997, ∼9% of women had no reduction in pelvic pain and 50% reported pelvic pain at the end of the follow-up period (8).

The main objectives of the present systematic review were to determine response rates to medical therapy, the frequency and extent of remaining endometriosis-associated pain symptoms, and the recurrence of pain symptoms after cessation of therapy. Furthermore, we set out to characterize the patient population for whom existing medical therapies do not provide relief from endometriosis-associated pain. Knowledge of these data is of central clinical importance because it helps to inform both health care professionals and patients and aids in managing patients’ expectations. In addition, such data form the basis for management decisions about unmet clinical needs and will assist in improving future clinical trial design.

## Materials and methods

### Search Strategy

**Figure 1:**
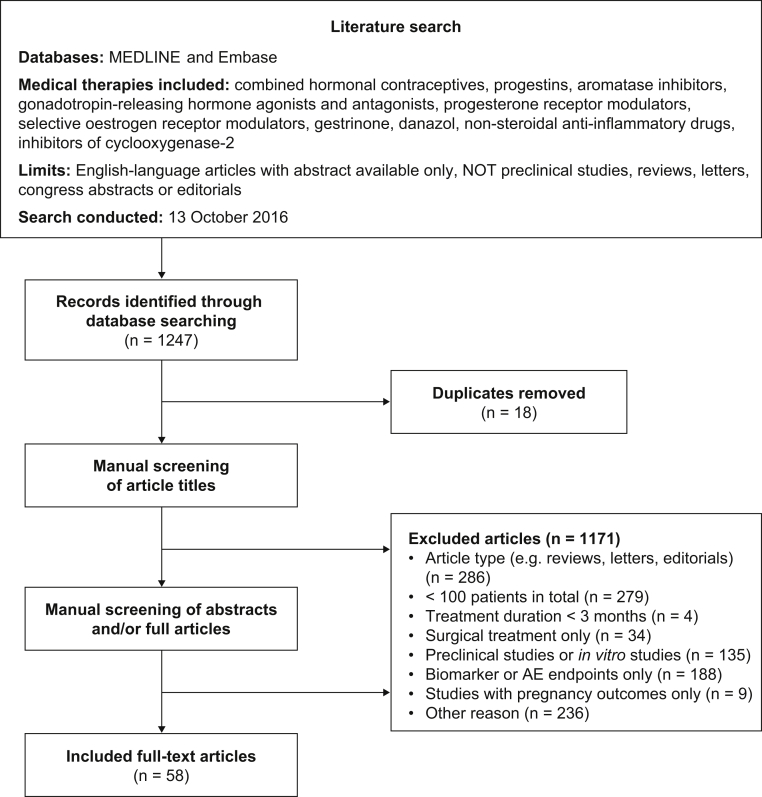
PRISMA (Preferred Reporting Items of Systematic Reviews and Meta-Analyses) flow diagram of the literature search and article selection process. AE = adverse events.

Medline and Embase were searched with the use of Ovid on October 13, 2016, to identify all studies reporting treatment response to medical therapy for endometriosis (Fig. 1). For details of the search strings used, see Supplemental Table 1 (Supplemental Tables 1–8 are available online at www.fertstert.org). The medical therapies included were danazol, gestrinone, combined hormonal contraceptives (CHCs), GnRH agonists, GnRH antagonists, progestins, mifepristone, aromatase inhibitors, selective estrogen receptor modulators, NSAIDs, and cyclooxygenase-2 inhibitors.

After removal of duplicates, all identified references were screened and categorized by two independent investigators. The exclusion criteria were: studies with <100 patients in total; treatment duration of <3 months; studies of surgical treatment only; preclinical research; articles reporting only data on biomarkers or adverse events; studies with pregnancy outcomes only; and inappropriate article type (e.g., reviews, letters, and editorials; Fig. 1). The protocol was registered with Prospero (CRD42015016633). The search was limited to English-language articles with available abstracts. No limit was set for the year of article publication. Records were initially screened based on title only; when possible, those meeting the exclusion criteria were excluded at that point. The remaining records were screened based on abstract and/or full article.

### Response to Treatment

From each article identified for inclusion, we extracted data for response rates related to endometriosis-associated pain for each treatment arm of the study. When available, we also extracted data for the placebo group. We collected information on the proportions of patients with no improvement in endometriosis-associated pain symptom severity (lack of response), with any pain remaining at the end of treatment (pain present at this point), and with recurrence of pain symptoms after treatment cessation. Information on the proportion of patients discontinuing therapy owing to adverse events or lack of efficacy was also obtained. Where data were available for more than one pain symptom, all values were extracted. Values were not extracted from articles if the data were presented only in graphic form.

Results for individual treatment arms were pooled according to the type of therapy. CHCs comprised COCs, vaginal ring, and contraceptive patch. Studies of the use of medical therapy after surgery were classified as a single group. Data were collected on treatment response, in terms of pain score reported before, during, and after treatment, and values on the 10-cm visual analog scale (VAS) assessed by the patients (0 cm representing no pain, 10 cm representing most severe pain). We also extracted data on the proportions of patients with an increase or no change during treatment in disease score, evaluated according to the revised American Society for Reproductive Medicine (ASRM) system, which is based on a clinician's assessment of endometrial implant size and location, degree of posterior cul-de-sac obliteration, and location and characteristics of adhesions. Finally, patient-reported ratings of treatment satisfaction considering overall well-being and quality of life, any adverse effects experienced, and convenience of treatment were evaluated using a 5-point Likert-type scale (very satisfied, satisfied, uncertain, dissatisfied, very dissatisfied). For each outcome, the range of values reported in all the included publications was extracted and the median calculated; data are presented as median (range) or as single values. Articles were examined for data that related treatment response to patient characteristics.

## Results

### Studies Meeting Eligibility Criteria

In total, 1,247 articles were identified (Fig. 1). Of these, 18 were duplicates and 1,171 were excluded after manual screening of article titles. After screening of abstracts and/or full articles, 58 studies met the eligibility criteria for the study (Supplemental Table 2). The main reasons for exclusion were article type (n = 286), insufficient number of patients (n = 279), studies with biomarker or adverse event outcomes only (n = 188), and other reasons (n = 236). Some articles were excluded on the basis of meeting multiple exclusion criteria.

### Characteristics of Studies

The characteristics of articles meeting the inclusion criteria are presented in Table 1. Most of the included articles (54 of 58) described clinical trials; only four reported observational data 11, 33, 42, 47. In most of the articles (48 of 58), endometriosis was diagnosed surgically. Women with all types of endometriosis were included in more than three-fourths of studies (44 of 58), and only the location and/or depth of endometriotic lesions were classified in three of 58 studies. One-fifth of studies (12 of 58) included only women with genital, pelvic, rectovaginal, or ovarian endometriosis. In nearly one-fourth of studies (14 of 58), surgery preceded medical therapy for all participants, and surgery preceded medical therapy in some participants in more than one-third of studies (20 of 58). No surgery preceded medical therapy in six studies, and surgical status was not described in 18 studies.

**Table 1:** Characteristics of articles in the study.

Therapy	No. of articles	Publication year(s)	Total no. of patients^a^	Treatment duration, mo	Study type	Funding source^b^
Danazol	3	1982–1998	481	4 to ≥6	Prospective cohort study (n = 2) 9, 10; retrospective study (n = 1) (11)	Nonindustry (n = 3)
Gestrinone	3	1995	702	6	RCT (n = 3) 12, 13, 14	Nonindustry (n = 3)
Mifepristone	1	2016	270	6	RCT (n = 1) (15)	Nonindustry (n = 1)
GnRH agonists	14	1988–2000	2,783	3–6	RCT (n = 12) 16, 17, 18, 19, 20, 21, 22, 23, 24, 25, 26, 27; prospective cohort study (n = 2) 28, 29	Industry (n = 8); partial industry (n = 3); nonindustry (n = 3)
GnRH agonists plus add-back therapy	4	1998–2016	738	5–12	RCT (n = 3) 30, 31, 32; prospective cohort study (n = 1) (33)	Industry (n = 1); partial industry (n = 1); nonindustry (n = 2)
Progestins	14	2000–2016	2,694	3–12	RCT (n = 8) 34, 35, 36, 37, 38, 39, 40, 41; prospective cohort study (n = 5) 42, 43, 44, 45, 46; retrospective study (n = 1) (47)	Industry (n = 7); partial industry (n = 2); nonindustry (n = 5)
CHCs^c^	3	2008–2013	555	12–23	RCT (n = 1) (48); prospective cohort study (n = 2) 49, 50	Nonindustry (n = 3)
GnRH antagonists	3	2013–2014	460	2–5.5	RCT (n = 3) 51, 52, 53	Industry (n = 3)
Medical and surgical treatment	13	1992–2014	3,198	3–24	RCT (n = 7) 54, 55, 56, 57, 58, 59, 60; prospective cohort study (n = 2) 61, 62; prospective case-control study (n = 2) 63, 64; retrospective study (n = 2) 65, 66	Nonindustry (n = 11); partial industry (n = 2)

*Note:* RCT, randomized controlled trial.

a Number of patients included in the efficacy analysis.

b Studies were classified as partial industry funding if a commercial organization provided the study drug or this was stated in the article.

c Combined hormonal contraceptives (CHCs) includeded combined oral contraceptives, vaginal ring, and contraceptive patch.

The most common classes of therapy included in the study were GnRH agonists and progestins (14 articles each). During screening of abstracts, three clinical studies were identified that reported on the use of NSAIDs to treat women with endometriosis; however, all were excluded on the basis of low patient numbers. Despite being widely prescribed for women with endometriosis, CHCs were the focus of only three eligible articles. These comprised one RCT comparing COCs with placebo (48), one prospective cohort study of continuous and cyclic COC regimens after surgical treatment (49), and a patient preference study of the contraceptive ring and patch (50). The treatments reported varied according to date of publication, reflecting the shift in medical therapy from danazol and gestrinone (publication years 1982–1998) and GnRH agonists (publication years 1988–2000) to progestins (publication years 2000–2016) and CHCs (publication years 2008–2013). Most studies of drugs for which approval for endometriosis treatment was sought, including GnRH agonists, GnRH antagonists, and progestins, were funded by industry, whereas none of the three studies of CHCs received industry funding.

Most articles described two or more treatment groups; there were 125 treatment arms in total. Most studies (79.3%) included assessment of endometriosis-associated pain. The most commonly used methods were 4-point subjective scales (22.4%), VAS score (22.4%), and the Biberoglu and Behrman score or modified versions thereof (15.5%) (67). Three studies (5.2%) used more than one method to measure pain symptoms. Data for the response of pain symptoms to treatment were presented in only 29 of the 58 articles identified. Information was available in different studies for pelvic tenderness and the following pain symptoms: dysmenorrhea, pelvic pain, nonmenstrual pelvic pain, dyspareunia, dyschezia, and abdominal pain. Separate data on induration were reported in only four studies and therefore were not extracted. No studies reported on the entire spectrum of endometriosis-associated pain symptoms; 25 studies included data on three or more pain symptoms.

### Lack of Response: Patients Reporting No Reduction in Endometriosis-Associated Pain Symptom Severity

**Figure 2:**
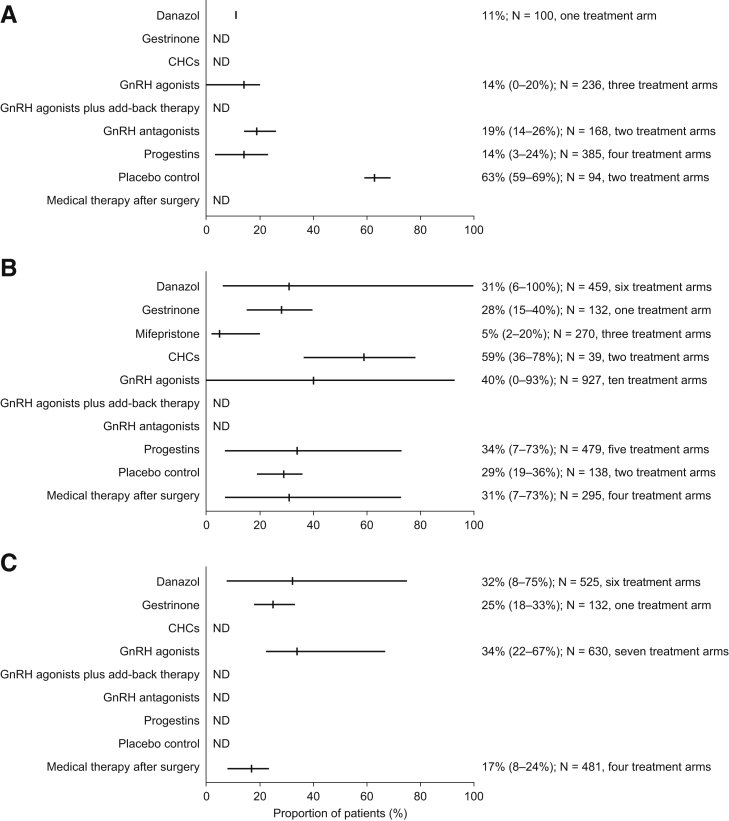
Response to therapy and symptom recurrence after treatment cessation. Proportions of patients with (**A**) no reduction in pain symptoms, (**B**) pain symptoms remaining at end of treatment, and (**C**) recurrence of pain symptoms after treatment cessation. Results are presented as median (range). ND = no data; CHC = combined hormonal contraceptive.

Lack of response to treatment (no reduction in endometriosis-associated pain symptoms during treatment, assessed by patient interview or symptom severity scoring [0 or <1-point decrease on a 4-point scale]) was reported in six studies (Fig. 2A; Supplemental Table 3) 9, 16, 17, 34, 35, 51. In four of these studies, pain symptom severity was patient reported, and in the other two it was physician reported. A further three studies presented these data in graphic form only and were not included in the analysis 36, 37, 68. The median proportion of patients with lack of response was highest for those treated with GnRH antagonists (19%, range 14%–26%, two treatment arms) (51). Among women receiving danazol, GnRH agonists, or progestins, the median proportions of individuals experiencing no improvement were 11% (one treatment arm) (9), 14% (range 0%–20%, three treatment arms) 16, 17, and 14% (range 3%–24%, four treatment arms) 34, 35, 51, respectively. Only two studies reported data for women in a placebo control group 17, 35. Nearly two-thirds of those women (median 63%, range 59%–69%, two treatment arms) had no response to placebo treatment 17, 35.

### Persistence of Any Endometriosis-Associated Pain Symptoms at the End of Medical Treatment

Persistence of endometriosis-associated pain symptoms at the end of therapy (i.e., patient-reported presence of any pain symptoms at the end of treatment) was described in 14 studies (Fig. 2B; Supplemental Table 4) 12, 15, 16, 18, 19, 20, 35, 38, 42, 47, 50, 54, 61, 62. One additional study (21) presented these data in graphic form only and was not included in the analysis. The median proportion of women with any pain symptoms remaining after medical therapy varied between 5% for women treated with mifepristone (range 3%–20%, three treatment arms) (15) to 59% (range 36%–78%) for women who received CHCs (two treatment arms: vaginal ring supplying ethinyl estradiol and etonogestrel or transdermal ethinyl estradiol and norelgestromin) (50).

Generally, there was a wide variation in the proportions of patients with pain symptoms remaining at the end of treatment. The widest range (6%–100%, median 31%, six treatment arms) was observed for patients treated with danazol 12, 18, 19, 20, 69. There was a similarly wide range in patients who received GnRH agonists (0%–93%, median 40%, ten treatment arms) 16, 18, 19, 20, 38, 69. In addition, more than one-third of women who received progestins (median 34%, range 7%–73%, five treatment arms) 35, 38, 42, 47 and nearly one-third of patients who received GnRH analogue therapy after surgery (median 31%, range 7%–73%, four treatment arms) had pain symptoms remaining at the end of medical treatment 50, 61, 62. More than one-fourth of women who were treated with gestrinone (median 28%, range 15%–40%, one treatment arm) (12) and more than one-fourth of those who received placebo (median 29%, range 20%–36%, two treatment arms) reported pain symptoms remaining at the end of the treatment 15, 35.

### Recurrence of Endometriosis-Associated Pain Symptoms after Treatment Cessation

The proportion of patients with recurrence of endometriosis-associated pain after treatment cessation (i.e., patient-reported presence of any pain symptoms at the end of a follow-up period after the end of treatment) was recorded in nine studies (Fig. 2C; Supplemental Table 5) 9, 12, 18, 20, 22, 49, 54, 55, 69. One further study (56) presented such data in graphic form only and was not included in the analysis. Approximately one-third of patients treated with danazol (median 32%, range 8%–75%, six treatment arms) 9, 12, 18, 20, 22, 69 or GnRH agonists (median 34%, range 22%–67%, seven treatment arms) 18, 20, 22, 54, 69 had recurrence of pain symptoms after median follow-up periods of 12 months and 9 months, respectively. The median proportion of patients with recurrence of pain symptoms 12 months after the end of gestrinone treatment was 25% (range 18%–33%, one treatment arm) (12). A median of 17% of women (range 8%–24%, four treatment arms) experienced recurrence of pain symptoms after GnRH agonist or CHC (cyclic or continuous oral drosperinone and ethinylestradiol) therapy following surgery 49, 54, 55.

### Response of Endometriosis-Associated Pain Symptoms to Therapy (VAS Score)

**Figure 3:**
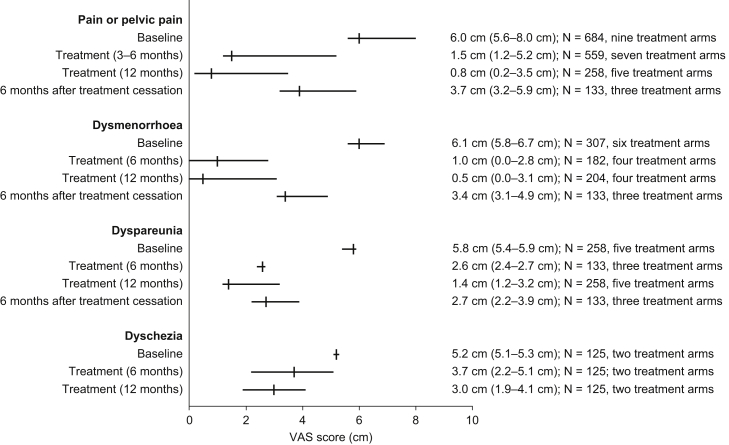
Visual analog scale (VAS) scores for pain symptoms at baseline and during and after treatment for patients receiving medical therapy. Results are presented as median (range).

Patient-reported VAS data were available from six studies 30, 34, 39, 42, 43, 48; an additional four articles 40, 44, 45, 46 presented VAS data in graphic form only and were not included in the analysis. Owing to the paucity of data, values were pooled for all medical therapies; patients treated with medical therapy after surgery were not included. Data for individual medical therapies are presented in Supplemental Table 6. Median VAS scores at baseline for pain or pelvic pain, dysmenorrhea, dyspareunia, and dyschezia were 6.0 cm (range 5.6–8.0 cm, nine treatment arms) 30, 34, 39, 42, 43, 6.1 cm (range 5.8–6.7 cm, six treatment arms) 30, 43, 48, 5.8 cm (5.4–5.9 cm, five treatment arms) 30, 43, and 5.2 cm (5.1–5.3 cm, two treatment arms) (43), respectively (Fig. 3).

During treatment, median VAS scores for pain or pelvic pain decreased to 1.5 cm (range 1.2–5.2 cm, seven treatment arms) after 3–6 months 30, 34, 39, 41, 42 and decreased further to 0.8 cm (range 0.2–3.5 cm, five treatment arms) after 12 months 30, 43. The median VAS score for dysmenorrhea decreased to 0.5 cm (range 0.0–3.1 cm, four treatment arms) after treatment for 12 months 30, 43. At the end of 12 months’ treatment, median VAS scores for dyspareunia and dyschezia had reduced by a lesser extent than the other pain symptoms, with values of 1.4 cm (range 1.2–3.2 cm, five treatment arms) (30) and 3.0 cm (range 1.9–4.1 cm, two treatment arms) (43), respectively.

Six months after treatment cessation, the median VAS score for pain or pelvic pain increased to 3.7 cm (range 3.2–5.9 cm, three treatment arms) (30). Similarly, median VAS scores for dysmenorrhea and dyspareunia both increased to 3.4 cm and 2.7 cm (ranges 3.1–4.9 cm and 2.2–3.9 cm, respectively) 6 months after treatment cessation (30).

For patients who received CHCs, the median VAS score for pelvic pain at baseline was 6.0 cm (range 5.6–6.3 cm, two treatment arms) 30, 43. During treatment, it decreased to 1.9 cm (one treatment arm) after 6 months (30) and to 2.2 cm (range 0.8–3.5 cm, two treatment arms) after 12 months 30, 43. Six months after the end of treatment, it returned to the baseline level of 5.9 cm (one treatment arm) (30).

### Proportion of Patients with an Increase or No Change in Disease Score, Based on the Revised ASRM System

In total, ten studies reported data on the lack of treatment response in terms of an increase or no change in disease score evaluated according to the revised ASRM system (based on a clinician's assessment of endometrial implant size and location, degree of posterior cul-de-sac obliteration, and location and characteristics of adhesions) (Supplemental Table 7) 9, 13, 18, 21, 23, 24, 28, 35, 61, 63. The median proportions of patients whose disease score did not decrease with medical therapy ranged from 17% to 45% 9, 13, 18, 21, 23, 24, 28, 35, 61, 63.

### Discontinuation due to Lack of Efficacy or Adverse Events

The highest proportion of patients discontinuing treatment owing to lack of efficacy or adverse events was for those treated with gestrinone (median 16%, range 11%–21%; Supplemental Table 8) 12, 13. Among patients treated with progestins, danazol, GnRH agonists, or GnRH antagonists, a median of 5%–9% stopped treatment due to adverse events or lack of efficacy (median duration of study was 6 months in each case) 9, 12, 13, 16, 17, 18, 19, 21, 23, 24, 25, 28, 34, 36, 37, 39, 42, 43, 44, 45, 47, 51, 52, 53, 57, 61. A higher proportion of women who received GnRH agonists with add-back therapy (median 12%, range 10%–15%) discontinued treatment during a 5-month period (33). Over a median study duration of 12 months, a median of 8% (range 5%–24%) of women discontinued CHCs because of adverse events or lack of efficacy 43, 45, 48, 49, 50, 56, 58. It was not possible to analyze treatment discontinuation due to lack of efficacy or adverse events separately because of the way in which findings were reported in the included studies.

### Patient Satisfaction

Patient-reported ratings of treatment satisfaction considering overall well-being and quality of life, any adverse effects experienced, and convenience of treatment were evaluated in only five studies 43, 45, 46, 47, 50. These studies showed that a median of 43% of women who received CHCs (range 36%–71%, four treatment arms) 43, 45, 50 and a median of 62% of women who received a progestin (range 59%–72%, five treatment arms) 43, 45, 47 were satisfied with their treatment. Among women who received progestin therapy, a median of 27% were dissatisfied with treatment 43, 45, 46, 47, compared with a median of 34% who had endometriosis-associated pain remaining at the end of treatment 35, 38, 42, 47. In contrast, a smaller proportion of women were dissatisfied with CHC treatment (median 31%) 43, 45, 50 than had pain remaining at the end of CHC treatment (median 59%) (50). In the only two studies that reported both of these outcomes for the same patient groups, among those who received CHCs a median of 28% were dissatisfied with treatment and a median of 59% had persistent pain (50), and among women who were treated with progestins a median of 27% were dissatisfied with therapy and a median of 34% had persistent pain (47).

### Patients Who Do Not Respond to Treatment

None of the articles examined provided information about the characteristics of patients who had no response to treatment (those who experienced either no reduction in endometriosis-associated pain symptoms or persistent symptoms during treatment).

## Discussion

Endometriosis has long been recognized as a hormone-dependent disease affecting millions of women worldwide. The cardinal symptom is pain, which often significantly impairs the lives of affected women, their partners, and families, with substantial socioeconomic ramifications (70). Endometriotic lesions are commonly treated by means of surgical ablation or excision, or by medical suppression of endogenous hormone levels. Daily clinical practice provides plentiful evidence of the deficiencies of current therapies, such as limited efficacy, high rates of symptom recurrence, and significant side-effects from treatment. Published data reflecting clinical experience, however, are sparse. The present systematic review comprehensively assessed the response rates of patients to medical therapy for endometriosis-associated pain. The available data strongly suggest that, regardless of the type of hormonal treatment used, many women remain symptomatic during or after treatment or have high symptom recurrence rates after therapy cessation. In addition, some women stop treatment owing to lack of efficacy or intolerable side-effects.

In most of the studies included in this review, endometriosis was diagnosed surgically. One study described the potential effectiveness of medical therapy (injection of the GnRH agonist leuprolide) in selected women with clinically suspected endometriosis before laparoscopic confirmation (17). Early implementation of medical treatment may prevent disease progression and tissue damage in many women without the need for invasive procedures (71).

For most women, endometriosis-associated pain symptoms are reduced by treatment, but our study showed that 5%–59% had pain remaining at the end of treatment. Furthermore, 11%–19% of women with endometriosis derived no pain relief at all from medical therapy. In more than one-half of the studies, however, some or all participants underwent surgery before the initiation of medical therapy, so it is possible that persistent pain may have resulted from surgical complications, such as adhesions, in some women. As expected, women in the placebo arms of clinical studies were least likely to experience a reduction in pain symptoms during treatment, although some patients who received placebo did report some benefit. This may be due to the influence of cognitive factors, such as expectation on nociceptive processing (72), and the inhibition of nociception by placebo treatment reducing neural responses to pain stimuli in the brain and thus decreasing pain sensation (73). Clear conclusions, however, can not be made on lack of response (in terms of both persistent pain and lack of reduction in pain symptom severity) to the therapies examined, because it was not assessed consistently in the included studies.

Even when medical therapy does provide relief of symptoms, recurrence of pain symptoms after treatment cessation is common, reported in 17%–34% of treated women. The continuation of treatment to obtain sustained symptom relief may, however, be limited by drug intolerance or increased exposure to the risk of adverse events. Prospective data on the long-term efficacy and safety of medical therapies for the different types of endometriosis are needed to determine optimal and maximum treatment durations.

VAS scores of endometriosis pain symptoms were reported by six studies and provide a consistent measure of treatment effectiveness. For pain or pelvic pain, the median VAS score decreased from 6.0 cm to 0.8 cm after 12 months' treatment, but it rose to 3.7 cm by 6 months after treatment cessation. Similarly, the median VAS score for dysmenorrhea, which dropped from 6.1 cm to 0.5 cm after 12 months’ treatment, increased to 3.4 cm after a 6-month post-treatment follow-up period. These results demonstrate a substantial reduction in pain symptoms during medical therapy and their frequent recurrence after treatment cessation.

Surprisingly, median rates of discontinuation due to adverse events or lack of efficacy were consistently low across different therapies, with discontinuation rates of 5%–9% among patients treated with CHCs, progestins, danazol, GnRH agonists, or GnRH antagonists. Among patients who received GnRH agonists with add-back therapy, 12% discontinued treatment (33). For the synthetic steroid hormone gestrinone, this value was 16%; however, it may be difficult to draw meaningful comparisons between discontinuation rates for therapies that are self-administered daily and those that are administered by injection once every 12 weeks.

Limited data on patient-centered ratings of treatment satisfaction were reported in the included studies. They were evaluated in only five studies, which showed that a median of 61% of women who received CHCs or a progestin were satisfied with their treatment 43, 45, 46, 47, 50. Interestingly, in one study, the proportion of women who were dissatisfied with CHC treatment was approximately one-half that of women who had pain remaining at the end of CHC treatment (31% and 59%, respectively) (50). These results indicate that some women are satisfied with their treatment even if it does not completely relieve their pain. A meta-analysis comparing measures of treatment outcome in women with endometriosis found that, although the patient-reported VAS pain score correlates well with the Clinical Global Impression efficacy index, it accounts for only 28% of the variability between different scales measuring patient-reported treatment satisfaction (67). This demonstrates that both patient-reported pain and clinician-assessed treatment efficacy are not the only aspects of living with endometriosis that affect women's quality of life, and it highlights the need to assess both endometriosis-associated pain and health-related quality of life with the use of a disease-specific tool (67).

There are few published reports on patients' rates of response to medical therapy for endometriosis-associated pain, with most existing studies being conducted many years ago, narrow in scope, or based on patient surveys. A review of progestins from 1997 found that 9% of women had no reduction in pelvic pain at the end of 1.5–13.5 months' treatment, and 50% reported pelvic pain 2–12 months after treatment cessation (8). In another study, Vercellini et al. investigated treatment outcomes for women with rectovaginal endometriosis (74). They found that 60%–90% of patients reported either a considerable reduction in or complete relief from endometriosis-associated pain symptoms, and most had an improvement in health-related quality of life and/or were satisfied with their medical treatment (with danazol, a GnRH agonist, progestin, or an estrogen–progestin combination) (74). Only seven studies met the inclusion criteria for this analysis 50, 75, 76, 77, 78, 79, 80, however, and some were of limited quality and the authors rightly highlighted the risk of reporting bias (74). In an international cross-sectional survey of women with endometriosis receiving treatment in tertiary care centers, 60% reported current chronic pain despite receiving treatment (70). Furthermore, according to the results of a survey of patients’ lifetime experience conducted by the Endometriosis Association, many women discontinued medical therapy because of ineffectiveness (range 15.6%–26.1%) or side-effects (range 10.0%–43.5%) (81).

A further objective of the present systematic review was to characterize patients who do not respond to existing medical therapies; however, none of the articles included in the present study reported the demographic characteristics (e.g., race/ethnicity, age) of those who did not respond to treatment. In addition, because women with all types of endometriosis were included without classification in most studies, it was not possible to correlate treatment response with type of endometriosis. Only one article reported on response to treatment stratified by baseline disease stage (9). Interestingly, a higher proportion of women with stage IV disease at baseline experienced symptomatic improvement following danazol therapy than women with stage I disease at baseline (100% and 80%, respectively). Symptom recurrence 5 years after the end of treatment, however, also occurred in more patients with stage IV disease at baseline (50%) than in patients with stage I disease at baseline (22%) (9). Further research is required to determine how disease stage affects treatment response.

No data on the type of pain experienced (e.g., nociceptive, neuropathic, or inflammatory pain) were available in the reviewed studies. This important information could be captured with the use of specifically developed and validated questionnaires, such as those published recently as part of the Endometriosis Phenome and Biobanking Harmonisation (EPHect) Project 82, 83. This multinational initiative developed standards for the collection of clinical and epidemiologic data relevant to endometriosis research, facilitating large-scale collaboration. Leading academic endometriosis centers have adopted the principles of the EPHect Project, and the questionnaires are freely available (http://endometriosisfoundation.org/ephect/). The collection and analysis of qualitative data on the background and medical history of women with endometriosis may improve understanding of the underlying pathologic processes of the disease and help in the development of novel treatment strategies and in the assessment of treatment outcome/effectiveness in clinical trials. Similarly, the Core Outcomes in Women's and Newborn Health initiative, which aims to harmonize outcome reporting in women's health research, will be a helpful tool in improving clinical trials in the future (84).

Our review has revealed several important limitations of existing studies of endometriosis treatment. Few articles provided data for the outcomes of interest. Owing to the limited availability of data, results from treatment arms were pooled by treatment type. Similarly, because of the way in which findings were reported, outcomes data for discontinuation due to lack of efficacy or adverse events were combined. In addition, although the present study had no restrictions on publication date and included a broad range of treatments, patient-centered ratings of treatment satisfaction were either absent or incompletely reported, and they were evaluated in only four studies 43, 45, 46, 47, 50. Furthermore, there was heterogeneity in the CHCs and progestins examined; we found a similar treatment heterogeneity and lack of consistently reported data in a systematic review of surgical treatment of endometriosis (Singh SS et al. A systematic review of endometriosis interventions: what is missing in the literature? Poster presented at the 2nd Congress of Society of Endometriosis and Uterine Disorders, Barcelona, Spain, May 12–14, 2016).

Only six articles reported the proportion of patients whose symptoms did not improve with treatment. However, those studies investigated responses to only four treatment types and measured different aspects of treatment response, including both endometriosis-associated pain and its impact on functional status. These were assessed with the use of a variety of methods, including patient- and physician-reported Biberoglu and Behrman scores, VAS scores, and patient interviews. This limited direct comparison of baseline pain scores and response to treatment, as well as the comparisons between studies and treatment types that could be made. It has been suggested that a general measure of endometriosis-associated pain, such as the VAS, may best reflect patient satisfaction with treatment, but there is no consensus on the best method of assessment 67, 85. The results of any instrument measuring patient-reported pain or treatment satisfaction will, however, reflect the method of assessment (e.g., patient interview, rating scale) and the wording of the questions used (e.g., general or specific).

Recent guidelines on pain scoring in clinical trials in endometriosis suggest that the definition of a responder should be provided in each study, as should the definition of a clinically meaningful effect from the perspective of the patient (86). As highlighted in a recent review of current and future medical therapies for women with endometriosis, the patient should be able to quantify the purported benefits of therapy (5). Similarly, the Initiative of Methods, Measurement and Pain Assessment in Clinical Trials recommends that assessment of participant ratings of improvement should be considered in the design of chronic pain clinical trials (87).

It is well established that medical therapy for endometriosis is one of the pillars of the treatment of endometriosis-associated pain. In particular, CHCs and progestins are inexpensive and well tolerated alternatives to surgical treatment. During clinical trials, many therapies have been shown to produce a statistically significant reduction in endometriosis-associated pain in the overall study population. There are many women, however, for whom medical therapy does not provide sufficient or sustained relief from endometriosis-associated pain, or who may be unable to receive treatment owing to contraindication or tolerability. These data should be considered and explained to women to manage expectations.

Although there are significant issues and inconsistencies in the reporting of outcomes in the studies reviewed, some observations can be made. Endometriosis is a chronic disease that requires long-term therapy (1). The mechanisms of action of available treatments provide symptom relief only, and at the present there is no cure for the condition (71). Although current medical therapies suppress endometriosis symptoms, they are not effective in all women with endometriosis or they provide only limited symptom improvement. Those women who do not respond to existing therapies may benefit from new therapies with different mechanisms of action. There is evidence to suggest that in many women who do respond to therapy, symptoms return after cessation of treatment, even after short follow-up periods. Our study demonstrates that further research collecting robust data on pain and patient satisfaction is needed to evaluate the effectiveness of current medical therapies for endometriosis and thereby improve our understanding of their benefits and optimal usage. We also found that there remains an unmet clinical need among women with endometriosis for a specific disease-modifying therapy to provide long-term symptom relief that persists after the treatment period.

## Conclusion

Few studies of medical therapies for endometriosis report outcomes that are relevant to patients, and there is a lack of data on the characteristics of the population of patients whose symptoms do not respond to treatment. The use of standardized outcomes and sufficient patient sample sizes in studies of the efficacy of medical therapies for endometriosis are needed to generate robust data that would facilitate comparisons between studies and treatments. Recurrence of endometriosis-associated pain after treatment cessation is common, even after the short follow-up times reported in these studies. Endometriosis is a chronic condition, and patients require new medical therapies that provide long-term benefit, in terms of prevention of both disease progression and pain recurrence, that is sustained after treatment cessation.
