# Supplementary material for: Reevaluating response and failure of medical treatment of endometriosis: a systematic review
Source: Fertil Steril. 2017 Jul;108(1):125–36. doi: 10.1016/j.fertnstert.2017.05.004 (PMC5494290; doi:10.1016/j.fertnstert.2017.05.004)
Supplement: Supplementary Tables 1–8 [file mmc1.docx]

**Supplemental Table 1.** Summary of search of MEDLINE^®^ and Embase^®^, performed using Ovid^®^ on 13 October 2016.

| # | **Search string** | **Number of hits** |
| --- | --- | --- |
| 1 | ENDOMETRIOSIS/ | 49 624 |
| 2 | (adenomyo$ or endometriosis$).tw. | 50 491 |
| 3 | (adenomyo$ or endometrio$).tw. | 64 559 |
| 4 | chocolate cyst$.tw. | 328 |
| 5 | or/1-4 | 73 165 |
| 6 | CONTRACEPTIVES, ORAL/ | 64 134 |
| 7 | CONTRACEPTIVES, ORAL, SYNTHETIC/ | 47 027 |
| 8 | CONTRACEPTIVES, ORAL, COMBINED/ | 49 679 |
| 9 | (combin$ adj3 (oral$ or hormon$) adj3 (pill$ or contracept$)).tw. | 7418 |
| 10 | CONTRACEPTIVES, ORAL, HORMONAL/ | 50 147 |
| 11 | CONTRACEPTIVE RING/ | 5 |
| 12 | contraceptive ring.tw. | 212 |
| 13 | VAGINAL RING/ | 3226 |
| 14 | vaginal ring.tw. | 1446 |
| 15 | CONTRACEPTIVE PATCH/ | 153 |
| 16 | contraceptive patch$.tw. | 429 |
| 17 | PROGESTERONE/ | 142 210 |
| 18 | PROGESTERONE CONGENERS/ | 6517 |
| 19 | progesterone$.tw. | 162 130 |
| 20 | PROGESTINS/ | 34 273 |
| 21 | (progestin$ or progestogen$ or gestagen$).tw. | 36 492 |
| 22 | DYDROGESTERONE/ | 2124 |
| 23 | dydrogesterone$.tw. | 964 |
| 24 | NORETHINDRONE/ | 12 155 |
| 25 | (norethindrone$ or norethisterone$).tw. | 6487 |
| 26 | LEVONORGESTREL/ | 14 465 |
| 27 | levonorgestrel$.tw. | 8980 |
| 28 | MEDROXYPROGESTERONE 17-ACETATE/ | 20 982 |
| 29 | medroxyprogesterone$.tw. | 12 508 |
| 30 | depo.tw. | 4496 |
| 31 | dmpa.tw. | 2241 |
| 32 | DIENOGEST/ | 980 |
| 33 | dienogest.tw. | 940 |
| 34 | INTRAUTERINE DEVICES, MEDICATED/ | 18 093 |
| 35 | lng-ius.tw. | 1424 |
| 36 | ((intrauterine$ or intra uterine$) adj3 levonorgestrel$).tw. | 2874 |
| 37 | DANAZOL/ | 10 220 |
| 38 | danazol$.tw. | 5493 |
| 39 | GONADOTROPINS/ | 39 246 |
| 40 | gonadotrop?in$.tw. | 124 787 |
| 41 | GnRH$.tw. | 45 294 |
| 42 | GONADORELIN/ | 62 559 |
| 43 | gonadorelin$.tw. | 519 |
| 44 | BUSERELIN/ | 6297 |
| 45 | buserelin$.tw. | 2895 |
| 46 | GnRH/ | 62 559 |
| 47 | GOSERELIN/ | 7909 |
| 48 | goserelin$.tw. | 2169 |
| 49 | LEUPROLIDE/ | 12 471 |
| 50 | (leuprolide$ or leuprorelin$).tw. | 5120 |
| 51 | NAFARELIN/ | 1280 |
| 52 | nafarelin$.tw. | 593 |
| 53 | TRIPTORELIN/ | 6336 |
| 54 | triptorelin$.tw. | 1707 |
| 55 | ELAGOLIX/ | 48 |
| 56 | elagolix.tw. | 41 |
| 57 | DEGARELIX/ | 513 |
| 58 | degarelix.tw. | 449 |
| 59 | PROGESTERONE RECEPTOR MODULATOR/ | 574 |
| 60 | SELECTIVE PROGESTERONE RECEPTOR MODULATOR/ | 574 |
| 61 | PRM$.tw. | 8145 |
| 62 | SPRM$.tw. | 289 |
| 63 | PRM/ | 1 |
| 64 | progesterone receptor modulat$.tw. | 739 |
| 65 | selective progesterone receptor modulat$.tw. | 455 |
| 66 | PROGESTERONE RECEPTOR ANTAGONIST/ | 6 |
| 67 | progesterone receptor antagonist$.tw. | 647 |
| 68 | ULIPRISTAL ACETATE/ | 679 |
| 69 | ULIPRISTAL/ | 682 |
| 70 | ulipristal acetate.tw. | 602 |
| 71 | ulipristal.tw. | 693 |
| 72 | TELAPRISTONE/ | 15 |
| 73 | telapristone.tw. | 25 |
| 74 | MIFEPRISTONE/ | 17 079 |
| 75 | mifepristone.tw. | 6916 |
| 76 | AROMATASE INHIBITORS/ | 17 488 |
| 77 | aromatase inhibitor$.tw. | 15 082 |
| 78 | aromatase inhibit$.tw. | 15 499 |
| 79 | ANASTROZOLE/ | 8075 |
| 80 | anastrozole.tw. | 3919 |
| 81 | LETROZOLE/ | 9074 |
| 82 | letrozole.tw. | 5748 |
| 83 | EXEMESTANE/ | 5038 |
| 84 | exemestane.tw. | 2920 |
| 85 | ESTROGEN RECEPTOR MODULATOR/ | 1956 |
| 86 | estrogen receptor modulat$.tw. | 6442 |
| 87 | oestrogen receptor modulat$.tw. | 640 |
| 88 | SELECTIVE ESTROGEN RECEPTOR MODULATOR/ | 11 428 |
| 89 | SERM$.tw. | 5498 |
| 90 | selective estrogen receptor modulat$.tw. | 6060 |
| 91 | selective oestrogen receptor modulat$.tw. | 611 |
| 92 | ANTI-INFLAMMATORY AGENTS, NON-STEROIDAL/ | 138 607 |
| 93 | ((non-steroid$ or non-steroid$ or non steroid$) adj (anti-inflammatory or antiinflammatory) adj (agent$ or drug$)).tw. | 34 076 |
| 94 | NSAID$.tw. | 54 836 |
| 95 | IBUPROFEN/ | 50 027 |
| 96 | MEFENAMIC ACID/ | 6359 |
| 97 | FLURBIPROFEN/ | 8836 |
| 98 | DICLOFENAC/ | 41 017 |
| 99 | NAPROXEN/ | 27 381 |
| 100 | ETORICOXIB/ | 2430 |
| 101 | INDOMETACIN/ | 102 692 |
| 102 | (ibuprofen or mefenamic acid or naproxen or flurbiprofen or diclofenac or etoricoxib or indometacin).tw. | 63 675 |
| 103 | CYCLOOXYGENASE INHIBITORS/ | 24 451 |
| 104 | (cyclo-oxygenase$ or cyclooxygenase$).tw. | 91 980 |
| 105 | (Cox-2 or Cox2).tw. | 64 028 |
| 106 | or/6-105 | 979 013 |
| 107 | PAIN/ | 491 678 |
| 108 | pain$.tw. | 1 316 769 |
| 109 | PELVIC PAIN/ | 7959 |
| 110 | pelvic pain.tw. | 18 272 |
| 111 | CHRONIC PELVIC PAIN/ | 11 741 |
| 112 | chronic pelvic pain.tw. | 7661 |
| 113 | ((adenomyo$ or endometriosis$) adj2 pain$).tw. | 1255 |
| 114 | LESION/ | 265 |
| 115 | lesion$.tw. | 1 667 361 |
| 116 | endometriotic lesion$.tw. | 2911 |
| 117 | ENDOMETRIOMA/ | 25 587 |
| 118 | endoemtrioma$.tw. | 3 |
| 119 | DYSPAREUNIA/ | 9784 |
| 120 | dyspareunia.tw. | 8503 |
| 121 | DYSMENORRHEA/ | 13 409 |
| 122 | dysmenorrhea.tw. | 8406 |
| 123 | DYSCHEZIA/ | 84 198 |
| 124 | dyschezia.tw. | 636 |
| 125 | DYSURIA/ | 10 024 |
| 126 | dysuria.tw. | 8989 |
| 127 | or/107-126 | 3 084 674 |
| 128 | efficac$.tw. | 1 550 319 |
| 129 | effective.tw. | 2 502 887 |
| 130 | reduc$.tw. | 6 002 399 |
| 131 | respon$.tw. | 6 391 699 |
| 132 | 5 and 106 and 127 | 8276 |
| 133 | 132 and (128 or 129 or 130 or 131) | 3633 |
| 134 | limit 133 to english language | 3227 |
| 135 | limit 134 to abstracts | 3184 |
| 136 | remove duplicates from 135 | 2319 |
| 137 | limit 136 to "review" | 504 |
| 138 | 136 not 137 | 1815 |
| 139 | conference abstract.af. | 2 355 040 |
| 140 | 138 not 139 | 1445 |
| 141 | 140 and (mice or murine).tw. | 69 |
| 142 | 140 and rat.tw. | 55 |
| 143 | 140 and baboon.tw. | 7 |
| 144 | 140 and monkey.tw. | 3 |
| 145 | 140 and animal model$.tw. | 16 |
| 146 | 140 and rabbit$.tw | 6 |
| 147 | 140 and (in vitro or in vivo).m_titl. | 63 |
| 148 | or/141-147 | 198 |
| 149 | 140 not 148 | 1247 |

*Note:* Eighteen duplicates were identified and removed using EndNote.

**Supplemental Table 2.** Details of all studies that met the inclusion criteria.

| **Study** | **Treatment(s)** | **Surgical status of patients** | **Disease description** | **Age, years** | **Disease classification** | **Method of pain assessment** | **Baseline pain score for each cohort** | **Pain score at end of treatment^c^** |
| --- | --- | --- | --- | --- | --- | --- | --- | --- |
| Zupi *et al.* 2004 ([1](#_ENREF_1)) | LA depot 11.25 mg every 3 months;  LA depot 11.25 mg every 3 months/EE 25 µg transdermal/NE 5 mg;  EE 30 µg/gestodene 0.75 mg | Prior surgical treatment; all patients with recurrent symptoms | Endometriosis | 35.1–36.1 (mean) | rASRM stage III–IV (100%) | VAS (dysmenorrhea, dyspareunia, pelvic pain) | Pelvic pain  6.3–6.9 cm | Pelvic pain  0.3–0.8 cm |
| Donnez *et al.* 1989 ([2](#_ENREF_2)) | Buserelin IN 300 µg three times daily; buserelin SC implant 6 mg | Laparoscopically confirmed ovarian endometriosis | Ovarian endometriosis | 27.3–28.2 | AFS stage moderate or severe (100%) | ND | ND | ND |
| Henzl *et al.* 1988 ([3](#_ENREF_3)) | Nafarelin 200 µg twice daily;  nafarelin 400 µg twice daily; | Diagnostic laparoscopy within previous 3 months | Pelvic endometriosis | > 30 (60%) | AFS stage III–IV (45%) | 4-point scale (dysmenorrhea, dyspareunia, NMPP) | ND | ND |
| Alkatout *et al.* 2013 ([4](#_ENREF_4)) | Surgery plus LA depot 3.75 mg SC every month;  surgery;  LA depot 3.75 mg SC every month | No prior surgical treatment | Endometriosis | Inclusion criteria 18–44 | EEC stage II–III (47–60%) | ND | ND | ND |
| Bergqvist *et al.* 2000 ([5](#_ENREF_5)) | Goserelin 3.6 mg SC depot every 4 weeks; nafarelin 200 µg SC depot every 4 weeks | Diagnostic laparoscopy within previous 2 months | Endometriosis | 31 (median) | ND | 4-point scale (dysmenorrhea, dyspareunia, pelvic pain) | ND | ND |
| Vercellini *et al.* 2010 ([6](#_ENREF_6)) | Vaginal ring (EE 15 µg/ETG 120 µg);  patch (EE 20 µg/17-deacetyl norgestimate 120 µg) | Surgery within previous 12 months | Endometriosis | ≥ 30 (77–78%) | AFS stage III–IV (51–52%) | VAS (dysmenorrhea, deep dyspareunia, NMPP) | Dysmenorrhea  77–80 mm | Dysmenorrhea  22–35 mm; dyspareunia 22 mm |
| Vlahos *et al.* 2013 ([7](#_ENREF_7)) | Cyclic COC (EE 30 µg/DRSP 3 mg); continuous COC (EE 30 µg/DRSP 3 mg) | Surgery prior to treatment | Endometriosis | 27–28 (mean) | rAFS stage III–IV (89–90%) | 25-item questionnaire (dysmenorrhea, deep dyspareunia, NMPP) | ND | ND |
| Barbieri *et al.* 1982 ([8](#_ENREF_8)) | Danazol (400 mg oral daily) | Diagnostic laparoscopy | Endometriosis | 26–27 (mean) | Kistner stage III–IV (27%) | Subjective symptoms | ND | ND |
| Wheeler *et al.* 1992 ([9](#_ENREF_9)) | LA 3.75 mg IM depot every 4 weeks; danazol oral^a^ | Diagnostic laparoscopy within previous 4 months | Endometriosis | 29.8–31.0 (mean) | rAFS score 22.2–24.3 (mean) | B&B | ND | ND |
| Leone Roberti Maggiore *et al.* 2014 ([10](#_ENREF_10)) | Desogestrel 75 µg daily continuous;  vaginal ring (EE 15 µg/ETG 120 µg sequential) | Diagnosis by physical examination and MRI | Rectovaginal endometriosis infiltrating the rectum | 33.8–34.7 (mean) | ND | VAS (dysmenorrhea, non-menstrual CPP, dyschezia, deep dyspareunia) | Dysmenorrhea 6.4–6.7 cm; CPP 5.6–5.7 cm; dyschezia 5.1–5.3 cm; deep dyspareunia  5.4–5.8 cm | Dysmenorrhea 3.1 cm; CPP 2.9–3.5 cm; dyschezia 1.9–4.1 cm; deep dyspareunia  2.5–3.2 cm |
| Strowitzki *et al.* 2012 ([11](#_ENREF_11)) | Dienogest 2 mg oral;  LA 3.75 mg IM ‘standard dose’ | Diagnostic laparoscopy within previous 3 months or surgery within previous 12 months | Endometriosis | 30.6–31.0 (mean) | rAFS stage III–IV (42.9–47.5%) | B&B (pelvic pain, dysmenorrhea, dyspareunia) | ND | Reduction in pelvic pain 46.0–47.5 mm |
| Strowitzki *et al.* 2010 ([12](#_ENREF_12)) | Dienogest 2 mg oral;  placebo | Histologically confirmed endometriosis within previous 12 months | Endometriosis | 31.4–31.5 (mean) | rASRM stage (70.6–70.8%) | VAS | ND | Reduction of 27.4 mm from baseline^c^ |
| Harada *et al.* 2009 ([13](#_ENREF_13)) | Dienogest 1 mg twice daily oral;  buserelin IN 300 µg three times daily | Diagnosed by laparoscopy, laparotomy or MRI/ultrasound | Endometrioma | 33.5–33.8 (mean) | ND | Subjective non-menstrual pain symptoms | Total pain symptom score 5.7–5.9 | Total pain symptom score 2.4–2.5 |
| Strowitzki *et al.* 2010 ([14](#_ENREF_14)) | Dienogest 2 mg oral daily; LA 3.75 mg IM depot every 4 weeks | Diagnostic laparoscopy within previous 3 months or surgery within previous 12 months | Endometriosis | 30.6–31.0 (mean) | rAFS stage III–IV (42.9–47.5%) | VAS (endometriosis-associated pelvic pain) | Pelvic pain 57.9–60.2 mm | Pelvic pain 11.9–12.7 mm |
| Yang *et al.* 2014 ([15](#_ENREF_15)) | Surgery plus GnRH analogue^b^; surgery | Cystectomy performed as part of study | Ovarian endometriosis | < 35 (73.0%) | ND | ND | ND | ND |
| Roghaei *et al.* 2010 ([16](#_ENREF_16)) | Surgery plus letrozole 2.5 mg oral daily plus calcium 1 g daily plus vitamin D 800 IU daily; surgery plus danazol 600 mg oral daily plus calcium 1 g daily plus vitamin D 800 IU daily; surgery plus placebo plus calcium 1 g daily plus vitamin D 800 IU daily | Surgery performed as part of study | Endometriosis | 31.9–32.3 (mean) | ND | 11-item scale (dysmenorrhea, dyspareunia, CPP) | Dysmenorrhea 2.0–4.6;  dyspareunia 0.6–1.3;  CPP 0.5–1.1 | Dysmenorrhea 0.8–1.2;  dyspareunia 0.4–0.9; CPP 0.8–1.1 |
| Bulletti *et al.* 1996 ([17](#_ENREF_17)) | Surgery plus danazol 200 mg oral three times daily; surgery plus GnRH analogue^c^; surgery | Surgery performed as part of study | Endometriosis | 27.3 (mean) | AFS stage I–III (100%) | ND | ND | ND |
| Harrison *et al.* 2000 ([18](#_ENREF_18)) | MPA 50 mg/day; placebo | Diagnosed by laparoscopy | Endometriosis | 31.5–31.7 (mean) | AFS stage III–IV (13.9–27.7%) | 4-point scale | ND | ND |
| Diamond *et al.* 2014 ([19](#_ENREF_19)) | Elagolix 150 mg oral daily; elagolix 250 mg oral daily; placebo | Diagnostic laparoscopy within previous 8 years | Endometriosis | 30.9–31.2 (mean) | rASRM stage III–IV (26.9–29.4%) | 11-point numerical rating scale (endometriosis-associated pain) | ND | Decrease from baseline 0.88–1.25 |
| Carr *et al.* 2013 ([20](#_ENREF_20)) | Elagolix 150 mg oral daily; placebo | Diagnostic laparoscopy within previous 8 years | Endometriosis | 33 (median) | rASRM stage III–IV (39.7–46.4%) | CPSSS | ND | Decrease from baseline: dysmenorrhea 1.13; NMPP 0.47; dyspareunia 0.61; cumulative pain score 0.55; CPSSS 4.5 |
| Carr *et al.* 2014 ([21](#_ENREF_21)) | Elagolix 75 mg oral twice daily; elagolix 150 mg oral daily; DMPA 104 mg SC every 12 weeks | Diagnostic laparoscopy within previous 7 years | Endometriosis | 31.4–32.4 (mean) | rASRM stage III–IV (33.3–45.3%) | CPSSS | ND | Decrease from baseline:  CPSSS 5.2–5.5 |
| Vercellini *et al.*1999 ([22](#_ENREF_22)) | Surgery plus goserelin 3.6 mg SC depot every 4 weeks; surgery plus expectant management | Surgery performed as part of study | Endometriosis | 30.0–30.1 (mean) | AFS stage III–IV (86.6–88.0%) | B&B (dysmenorrhea, deep dyspareunia, NMPP) | ND | ND |
| Reichel *et al.* 1992 ([23](#_ENREF_23)) | Goserelin 3.6 mg SC depot every 4 weeks | Diagnosis by laparoscopy | Endometriosis | 30.2 (mean) | rAFS stage III–IV (42.5%) | 4-point scale (dysmenorrhea, dyspareunia, pelvic pain) | ND | ND |
| Moghissi *et al.* 1998 ([24](#_ENREF_24)) | Goserelin 3.6 mg SC depot every 4 weeks; goserelin 3.6 mg SC depot every 4 weeks plus estrogen 0.3 mg plus MPA 5 mg; goserelin 3.6 mg SC depot every 4 weeks plus estrogen 0.625 mg plus MPA 5 mg | Diagnosis by laparoscopy within previous 2 years; surgery may have been performed | Endometriosis | 29.4–30.7 (mean) | ND | Total pelvic symptom score | ND | Decrease from baseline in total pelvic symptom score: 3.5–4.4 |
| Miller *et al.* 1998 ([25](#_ENREF_25)) | Danazol 400–800 mg oral daily; GnRH analogue^b^ | Diagnosis by laparoscopy | Endometriosis | 34.4 (mean) | rAFS stage III–IV (31%) | 4-point scale (dysmenorrhea, dyspareunia, pelvic pain) | ND | ND |
| Sesta *et al.* 2007 ([26](#_ENREF_26)) | Surgery plus triptorelin or LA 3.75 mg SC every 4 weeks; surgery plus COC (EE 30 µg and gestoden 0.75 mg); surgery plus dietary treatment; surgery | Surgery performed as part of study | Endometriosis | 29.0–31.0 (mean) | rAFS stage III–IV (100%) | VAS | Dysmenorrhea 7.7–8.2 cm;  NMPP 8.0–8.5 cm;  deep dyspareunia 6.8–7.2 cm | Dysmenorrhea 5.5–6.4 cm;  NMPP 4.7–6.2 cm;  deep dyspareunia 4.3–5.0 cm |
| Szendei *et al.* 2005 ([27](#_ENREF_27)) | Surgery plus GnRH analogue followed by surgery; surgery plus GnRH analogue followed by surgery plus COC^b^ | Surgery performed as part of study | Endometriosis | ND | ND | Short-form McGill pain questionnaire (dysmenorrhea, dyspareunia, pelvic pain) | ND | ND |
| Hornstein *et al.* 1998 ([28](#_ENREF_28)) | LA 3.75 mg SC every 4 weeks; LA 3.75 mg SC every 4 weeks plus NE acetate 5 mg; LA 3.75 mg SC every 4 weeks plus NE acetate 5 mg plus equine estrogens 0.625 mg; LA 3.75 mg SC every 4 weeks plus NE acetate 5 mg plus equine estrogens 1.25 mg | Diagnosis by laparoscopy within previous 12 months | Endometriosis | 27.9–29.0 (mean) | AFS stage moderate or severe (14–26%) | B&B (dysmenorrhea, pelvic pain) | ND | Decrease from baseline:  dysmenorrhea 1.7–1.9;  pelvic pain 0.6–0.9;  pelvic tenderness 0.7–0.8 |
| Seracchioli *et al.* 2010 ([29](#_ENREF_29)) | Surgery; surgery plus cyclic COC (EE 20 µg gestodene 0.075 mg); surgery plus continuous COC (EE 20 µg gestodene 0.075 mg) | Cystectomy performed as part of study | Ovarian endometriosis | 28.6–30.1 (mean) | AFS stage III–IV (100%) | ND | ND | ND |
| Seracchioli *et al.* 2010 ([30](#_ENREF_30)) | Surgery; surgery plus cyclic COC (EE 20 µg gestodene 0.075 mg); surgery plus continuous COC (EE 20 µg gestodene 0.075 mg) | Cystectomy performed as part of study | Ovarian endometriosis | 28.7–30.2 (mean) | AFS stage III–IV (100%) | VAS (dysmenorrhea, dyspareunia, CPP) | ND | ND |
| Momoeda *et al.* 2009 ([31](#_ENREF_31)) | Dienogest 1 mg oral twice daily | Diagnosis by laparoscopy or ultrasound | Endometriosis | 34.1 (mean) | ND | VAS (lower abdominal pain,  lumbago) | ND | Decrease from baseline:  lower abdominal pain 28.4 mm;  lumbago 19.8 mm |
| Harada *et al.* 2008 ([32](#_ENREF_32)) | Cyclic COC (EE 35 µg/NE 1 mg); placebo | Diagnosis by laparoscopy or ultrasound/MRI | Endometriosis | 31.5–31.7 (mean) | ND | VAS and VRS | VAS: pain 58.7; NMPP 27.5  VRS: pain 4.4 | VAS: pain 18.6; NMPP 19.1  VRS: pain 2.4 |
| Bromham *et al.* 1995 ([33](#_ENREF_33)) | Danazol 200 mg oral twice daily; gestrinone 2.5 mg oral twice weekly | Diagnosis by laparoscopy | Endometriosis | ND | AFS stage severe or extensive (11–19%) | 4-point scale (dysmenorrhea, dyspareunia, lower abdominal pain) | ND | ND |
| NEET Group 1992 ([34](#_ENREF_34)) | Nafarelin 200 µg IN twice daily; danazol 200 mg oral three times daily | Diagnosis by laparoscopy or laparotomy at admission | Endometriosis | 31–45 (61%) | AFS stage III–IV (39%) | 4-point scale (dysmenorrhea, dyspareunia, NMPP) | ND | ND |
| Rolland *et al.* 1990 ([35](#_ENREF_35)) | Nafarelin 200 µg IN twice daily; danazol 200 mg oral twice daily | Diagnosis by laparoscopy or laparotomy | Endometriosis | Inclusion criteria 18–45 | ND | 4-point scale (dysmenorrhea, dyspareunia, pelvic pain, pelvic tenderness) | ND | ND |
| Agarwal *et al.* 1997 ([36](#_ENREF_36)) | Nafarelin 200 µg IN twice daily; LA 3.65 mg IM monthly | Diagnosis by laparoscopy or laparotomy within previous 18 months | Endometriosis | 29.8–31.7 (mean) | Endometriosis score 11.2–11.7 | 4-point subjective scale (dysmenorrhea, dyspareunia, pelvic pain, tenderness) | Dysmenorrhea 1.9–2.0;  dyspareunia 1.1–1.2;  pelvic pain 1.7;  tenderness 1.3–1.5 | Dysmenorrhea 0.3–0.4;  dyspareunia 0.7–0.8;  pelvic pain 0.7–0.8;  tenderness 0.5–0.6 |
| Shaw *et al.* 1992 ([37](#_ENREF_37)) | Goserelin 3.6 mg SC every 4 weeks; danazol 200 mg oral four times daily | Diagnosis by laparoscopy | Endometriosis | ND | ND | ND | ND | ND |
| Adamson *et al.* 1994 ([38](#_ENREF_38)) | Nafarelin 200 µg IN twice daily; nafarelin 400 µg IN twice daily; danazol 400 mg oral daily | Diagnosis by laparoscopy | Endometriosis | ND | ND | 4-point subjective scale (dysmenorrhea, dyspareunia, NMPP) | ND | ND |
| Lee *et al.* 2010 ([39](#_ENREF_39)) | Surgery plus GnRH agonist^b^; surgery plus GnRH agonist followed by COC^b^ | Histologically confirmed endometriosis; cystectomy performed as part of study | Ovarian endometriosis | 29.3–30.1 (mean) | ASRM stage III–IV (100%) | ND | ND | ND |
| Morotti *et al.* 2014 ([40](#_ENREF_40)) | Desogestrel 150 µg plus EE 20 µg; desogestrel 75 µg | Diagnosis by examination and ultrasound | Rectovaginal endometriosis | 29.7–30.4 (mean) | ND | VAS | ND | ND |
| Hornstein *et al.* 1995 ([41](#_ENREF_41)) | Nafarelin 200 µg IN twice daily for 3 months; nafarelin 200 µg IN twice daily for 6 months | Diagnosis by laparoscopy or laparotomy within previous 24 months | Endometriosis | 31.0–31.3 (mean) | Endometriosis score 9.6–11.4 | 4-point subjective scale (dysmenorrhea, dyspareunia, NMPP) | Dysmenorrhea 1.9;  dyspareunia 1.6–1.8;  pelvic pain 1.6–1.8;  tenderness 1.4–1.6 | Dysmenorrhea 0.2–0.3;  dyspareunia 0.6–0.7;  pelvic pain 0.6–0.8;  tenderness 0.4–0.5 |
| Ling 1999 ([42](#_ENREF_42)) | LA 3.65 mg IM monthly; placebo | Diagnosis by non-invasive procedures | Endometriosis | 29.4–32.3 (mean) | ND | McGill pain questionnaire and  11-point analogue scale | 31.3–35.8 (McGill pain questionnaire) | 9.5 (McGill pain questionnaire) |
| Petraglia *et al.* 2012 ([43](#_ENREF_43)) | Dienogest 2 mg oral once daily | Diagnosis by laparoscopy | Endometriosis | Inclusion criteria 18–45 | Stages I–IV (ASRM classification) | VAS | 27.9 mm^c^ | 9.7 mm^c^ |
| Doberl *et al.* 1984 ([44](#_ENREF_44)) | Danazol 600 mg oral once daily; danazol 400–600 mg oral once daily | Diagnosis by laparoscopy; adhesiolysis and resection of endometriomas ≥ 3 cm | Endometriosis | 29.3–30.6 (mean) | ND | ND | ND | ND |
| Crosignani *et al.* 2006 ([45](#_ENREF_45)) | DMPA 104 mg SC every 3 months; LA 11.25 mg every 3 months | Diagnosis by laparoscopy | Endometriosis | 30.9–31.8 (mean) | ND | Composite of modified B&B score | ND | Mean composite score improvement from baseline 6.1–6.6 |
| Schlaff *et al.* 2006 ([46](#_ENREF_46)) | DMPA 104 mg SC every 3 months; LA 11.25 mg every 3 months | Diagnosis by laparoscopy within previous 42 months | Endometriosis | 29.2–32.1 (mean) | ND | Composite of modified B&B score (dysmenorrhea, dyspareunia, pelvic pain, tenderness, induration) | 10.0–10.3 | Mean composite score improvement from baseline 5.1–5.3 |
| Vercellini *et al.* 2012 ([47](#_ENREF_47)) | Surgery; NE acetate 2.5 mg oral daily | Prior surgical treatment for rAFS stage III–IV endometriosis within previous 24 months | Endometriosis | ≥ 30 (84%) | rAFS stage III–IV (100%) | VAS (dysmenorrhea, deep dyspareunia, dyschezia) | 89 mm | 39–51 mm |
| Gokmen *et al.* 1996 ([48](#_ENREF_48)) | Triptorelin 3.75 mg IM every 4 weeks | Diagnosis by laparoscopy | Endometriosis | 28.4 (mean) | rAFS stage ≥ II (100%) | ND | ND | ND |
| Gerhard *et al.* 1992 ([49](#_ENREF_49)) | Surgery plus LA 3.75 mg SC every month | Diagnosis by laparoscopy within previous 3 months | Endometriosis | 30 (mean) | rAFS stage III–IV (45.2%) | ND | ND | ND |
| Halbe *et al.* 1995 ([50](#_ENREF_50)) | Danazol 400–600 mg oral twice daily; gestrinone 2.5 mg oral twice weekly | Diagnosis by laparoscopy | Endometriosis | 31–32 (mean) | ND | 4-point subjective scale (dysmenorrhea, dyspareunia, pelvic pain, pre-menstrual pain) | ND | Reduction from baseline:  dysmenorrhea 1.0–1.2;  dyspareunia 0.6–0.7  pelvic pain 0.5–0.6 |
| Bromham *et al.* 1995 ([51](#_ENREF_51)) | Danazol 200 mg oral twice daily; gestrinone 2.5 mg oral twice weekly | Diagnosis by laparoscopy | Endometriosis | ND | AFS stage severe or extensive (11–19%) | ND | ND | ND |
| Hornstein *et al.* 1997 ([52](#_ENREF_52)) | Surgery plus nafarelin 200 µg IN twice daily; surgery plus placebo | Surgery performed as part of study | Endometriosis | 30.4–31.1 (mean) | rAFS score 11.5–16.7 | B&B (dysmenorrhea, dyspareunia, pelvic pain) | Pain score 5.0 | Pain score: 1.9 |
| Kaser *et al.* 2012 ([53](#_ENREF_53)) | Surgery plus NE acetate 5–15 mg oral daily | Surgery performed as part of study | Endometriosis | 20.5 (mean) | rASRM stage III–IV (7.8%) | 0–10 Numerical Rating Scale | 5.0 (median) | 0 (median) |
| Rock *et al.* 1993 ([54](#_ENREF_54)) | Goserelin 3.6 mg SC every 4 weeks; danazol 400 mg oral twice daily | Diagnosis by laparoscopy or laparotomy | Endometriosis | 29.7–30.4 (mean) | rAFS stage III–IV (40.1–47.7%) | 4-point subjective scale (dysmenorrhea, dyspareunia, pelvic pain) | ND | ND |
| Luisi *et al.* 2015 ([55](#_ENREF_55)) | Dienogest 2 mg oral once daily for up to 90 days | 49% had previous surgery for endometriosis | Endometriosis | 34.9 (mean) | ND | VAS | Pelvic pain, 80 mm | Pelvic pain, 52 mm |
| Carbonell  *et al.* 2016 ([56](#_ENREF_56)) | Mifepristone 2.5 mg, 5 mg, 10 mg oral once daily; placebo oral once daily | Diagnosis by laparoscopy | Endometriosis | 31.4–33.4 (mean ) | rAFS score 26.1–29.3 (mean) | ND | ND | ND |
| Vercellini *et al.* 2016 | NE acetate 2.5 mg oral once daily; dienogest 2 mg oral once daily | 66–72% had previous surgery for endometriosis | Endometriosis | 33.6–33.8 (mean) |  | 11-point numeric rating scale (dysmenorrhea, dyspareunia, pelvic pain, dyschezia); B&B | Dysmenorrhea 8; dyspareunia 7–8; pelvic pain 7; dyschezia 3–6 | Dysmenorrhea 0; dyspareunia 0; pelvic pain 0; dyschezia 0 |
| Tsai *et al.*  2016 ([57](#_ENREF_57)) | LA IM once four-weekly plus oral 1 mg estradiol valerate + 2.5 mg MPA once or twice daily | Diagnosis by surgery | Symptomatic endometriosis | 35.8–36.1 (mean) | rASRM stage III–IV | Modified B&B | 6.7–7.3 | 3.2–3.7 |

*Note:* ^a^Dose not specified; ^b^brand not specified; ^c^data not presented for patients who received placebo or expectant management.

AFS, American Fertility Society; ASRM, American Society for Reproductive Medicine; B&B, Biberoglu and Behrman; COC, combined oral contraceptive; CPP, chronic pelvic pain; CPSSS, composite pelvic signs and symptoms score (of dysmenorrhea, dyspareunia, non-menstrual pelvic pain, pelvic tenderness and pelvic induration); DMPA, depot MPA; DRSP, drosperinone; EE, ethinyl estradiol; EEC, endoscopic endometriosis classification; ETG, etonogestrel; GnRH, gonadotropin-releasing hormone; IN, intranasal; IM, intramuscular; IU, international unit; LA, leuprolide acetate; NE, norethisterone; NMPP, non-menstrual pelvic pain; MPA, medroxyprogesterone acetate; MRI, magnetic resonance imaging; ND, no data; NEET, Nafarelin Endometriosis European Trial; rAFS, revised AFS; rASRM, revised American Society for Reproductive Medicine; SC, subcutaneous; VAS, visual analogue scale; VRS, verbal rating scale.

**Supplemental Table 3.** Proportion of patients with no reduction in endometriosis-associated pain symptoms

| **Treatment** | **Study** | **Endometriosis-associated pain symptoms** | | | | | **Median (range)** |
| --- | --- | --- | --- | --- | --- | --- | --- |
|  |  | **Dysmenorrhea** | **Pelvic pain** | **Dyspareunia** | **Pelvic tenderness** | **Total symptoms** |  |
| Danazol | Barbieri *et al.* 1982 ([8](#_ENREF_8)) | ND | ND | ND | ND | 11% | 11% |
| GnRH agonists | Agarwal *et al.* 1997 ([36](#_ENREF_36)) | ND | ND | ND | ND | 13% | 14% (0–20%) |
|  | Agarwal *et al.* 1997 ([36](#_ENREF_36)) | ND | ND | ND | ND | 12% |  |
|  | Ling 1999 ([42](#_ENREF_42)) | 0% | 20% | 14% | 19% | ND |  |
| GnRH antagonists | Carr *et al.* 2014 ([21](#_ENREF_21)) | 14% | 14% | ND | ND | ND | 18.6%  (14–26.2%) |
|  | Carr *et al.* 2014 ([21](#_ENREF_21)) | 26.2% | 23.1% | ND | ND | ND |  |
| Progestins | Carr *et al.* 2014 ([21](#_ENREF_21)) | 13.7% | 23.5% | ND | ND | ND | 13.7%  (3.3–23.5%) |
|  | Strowitzki *et al.* 2010 ([14](#_ENREF_14)) | ND | 3.3% | ND | ND | ND |  |
|  | Strowitzki *et al.* 2010 ([14](#_ENREF_14)) | ND | 4.2% | ND | ND | ND |  |
|  | Harrison *et al.* 2000 ([18](#_ENREF_18)) | 17% | ND | ND | ND | ND |  |
| Placebo control | Ling 1999 ([42](#_ENREF_42)) | 59% | 63% | 61% | 64% | ND | 63%  (59–69%) |
|  | Harrison *et al.* 2000 ([18](#_ENREF_18)) | 69% | ND | ND | ND | ND |  |

*Note:* There were no data for patients who received aromatase inhibitors, combined hormonal contraceptives or GnRH agonists plus add-back therapy.

GnRH, gonadotropin-releasing hormone; ND, no data.

**Supplemental Table 4.** Proportion of patients with endometriosis-associated pain symptoms persisting at the end of treatment

| **Treatment** | **Study** | **Endometriosis-associated pain symptoms** | | | | | **Median (range)** |
| --- | --- | --- | --- | --- | --- | --- | --- |
|  |  | **Dysmenorrhea** | **Pelvic pain** | **Dyspareunia** | **Pelvic tenderness** | **Total symptoms** |  |
| Danazol | Bromham *et al.* 1995 ([33](#_ENREF_33)) | 48% | ND | 17% | ND | ND | 30.5%  (6–100%) |
|  | NEET Group 1992 ([34](#_ENREF_34)) | 100% | 25% | 7% | 23% | ND |  |
|  | Rolland *et al.* 1990 ([35](#_ENREF_35)) | ND | ND | ND | ND | 52% |  |
|  | Adamson *et al.* 1994 ([38](#_ENREF_38)) | 6% | 36% | 17% | ND | ND |  |
|  | Henzl *et al.* 1990 ([58](#_ENREF_58)) | ND | ND | ND | ND | 51% |  |
|  | Henzl *et al.* 1990 ([58](#_ENREF_58)) | ND | ND | ND | ND | 52% |  |
| Gestrinone | Bromham *et al.* 1995 ([33](#_ENREF_33)) | 40% | ND | 15% | ND | ND | 27.5%  (15–40%) |
| Mifepristone | Carbonell *et al.*  2016 ([56](#_ENREF_56)) | 4.9% | 19.6% | 10.9% | ND | ND | 4.9%  (1.9–19.6%) |
|  | Carbonell *et al.*  2016 ([56](#_ENREF_56)) | 5.7% | 11.9% | 1.9% | ND | ND |  |
|  | Carbonell *et al.*  2016 ([56](#_ENREF_56)) | 4.7% | 3.3% | 3.6% | ND | ND |  |
| Combined hormonal contraceptives | Vercellini *et al.* 2010 ([6](#_ENREF_6)) | 36% | 39% | 78% | ND | ND | 59% (36–78%) |
|  | Vercellini *et al.* 2010 ([6](#_ENREF_6)) | 64% | 54% | 73% | ND | ND |  |
| GnRH agonists | Strowitzki *et al.* 2012 ([11](#_ENREF_11)) | ND | ND | ND | ND | 72% | 40% (0–93%) |
|  | NEET Group, 1992 ([34](#_ENREF_34)) | 93% | 23% | 13% | 20% | ND |  |
|  | Rolland *et al.* 1990 ([35](#_ENREF_35)) | ND | ND | ND | ND | 43% |  |
|  | Agarwal *et al.* 1997 ([36](#_ENREF_36)) | 22% | 51% | 40% | 46% | ND |  |
|  | Agarwal *et al.* 1997 ([36](#_ENREF_36)) | 17% | 53% | 45% | 38% | ND |  |
|  | Adamson *et al.* 1994 ([38](#_ENREF_38)) | 2% | 43% | 32% | ND | ND |  |
|  | Adamson *et al.* 1994 ([38](#_ENREF_38)) | 0% | 35% | 31% | ND | ND |  |
|  | Henzl *et al.* 1990 ([58](#_ENREF_58)) | ND | ND | ND | ND | 46% |  |
|  | Henzl *et al.* 1990 ([58](#_ENREF_58)) | ND | ND | ND | ND | 53% |  |
|  | Henzl *et al.* 1990 ([58](#_ENREF_58)) | ND | ND | ND | ND | 43% |  |
| Progestins | Strowitzki *et al.* 2012 ([11](#_ENREF_11)) | ND | ND | ND | ND | 73% | 34.4%  (7–73%) |
|  | Harrison *et al.* 2000 ([18](#_ENREF_18)) | 7% | 15% | ND | ND | ND |  |
|  | Luisi *et al.* 2015 ([55](#_ENREF_55)) | ND | 41.6% | ND | ND | ND |  |
|  | Vercellini *et al.*  2016 ([59](#_ENREF_59)) | 18.6% | 29.1% | 45.3% | ND | ND |  |
|  | Vercellini *et al.*  2016 ([59](#_ENREF_59)) | 11.7% | 39.7% | 45.9% | ND | ND |  |
| Placebo control | Harrison *et al.* 2000 ([18](#_ENREF_18)) | 30% | 21% | ND | ND | ND | 28.8%  (19.1–36.1%) |
|  | Carbonell *et al.*  2016 ([56](#_ENREF_56)) | 36.1% | 28.8% | 19.1% | ND | ND |  |
| Medical therapy after surgery | Gerhard *et al.* 1992 ([49](#_ENREF_49)) | 7% | 26% | 17% | ND | ND | 31%  (7–73%) |
|  | Szendei *et al.* 2005 ([27](#_ENREF_27)) | 19.5% | 15.3% | 35.6% | ND | ND |  |
|  | Vercellini *et al.* 2010 ([6](#_ENREF_6)) | 53% | 41% | 35% | ND | ND |  |
|  | Vercellini *et al.* 2010 ([6](#_ENREF_6)) | 73% | 59% | 27% | ND | ND |  |

*Note:* Combined hormonal contraceptives comprised combined oral contraceptives, vaginal ring and patch. There were no data for patients who received aromatase inhibitors, GnRH antagonists or GnRH agonists plus add-back.

GnRH, gonadotropin-releasing hormone; ND, no data; NEET, Naferelin Endometriosis European Trial.

**Supplemental Table 5.** Proportion of patients with recurrence of pain symptoms after treatment cessation

| **Treatment** | **Study** | **Endometriosis-associated pain symptoms** | | | | | | **Follow-up time (months)** | **Median (range)** |
| --- | --- | --- | --- | --- | --- | --- | --- | --- | --- |
|  |  | **Dysmenorrhea** | **Pelvic pain** | **Dyspareunia** | **Pelvic tenderness** | **Abdominal pain** | **Total symptoms** |  |  |
| Danazol | Henzl *et al.* 1990 ([58](#_ENREF_58)) | ND | ND | ND | ND | ND | 22% | 6 | 32%  (8–75%) |
|  | Barbieri *et al.* 1982 ([8](#_ENREF_8)) | ND | ND | ND | ND | ND | 33% | 46 (median) |  |
|  | Wheeler *et al.* 1992 ([9](#_ENREF_9)) | 75% | 32% | ND | 8% | ND | ND | 12 |  |
|  | NEET Group 1992 ([34](#_ENREF_34)) | 59% | 34% | 16% | 26% | ND | ND | 12 |  |
|  | Adamson *et al.* 1994 ([38](#_ENREF_38)) | 50% | 50% | 30% | ND | ND | ND | 6 |  |
|  | Bromham *et al.* 1995 ([33](#_ENREF_33)) | 39% | ND | 13% | ND | 30% | ND | 12 |  |
| Gestrinone | Bromham *et al.* 1995 ([33](#_ENREF_33)) | 33% | ND | 18% | ND | 25% | ND | 12 | 25%  (18–33%) |
| GnRH agonists | Alkatout *et al.* 2013 ([4](#_ENREF_4)) | 28% | ND | 22% | ND | 26% | ND | 12 | 34%  (22–67%) |
|  | Wheeler *et al.* 1992 ([9](#_ENREF_9)) | 67% | 61% | ND | 33% | ND | ND | 12 |  |
|  | NEET Group, 1992 ([34](#_ENREF_34)) | 59% | 35% | 13% | 23% | ND | ND | 12 |  |
|  | Adamson *et al.* 1994 ([38](#_ENREF_38)) | 67% | 51% | 29% | ND | ND | ND | 6 |  |
|  | Adamson *et al.* 1994 ([38](#_ENREF_38)) | 64% | 55% | 35% | ND | ND | ND | 6 |  |
|  | Henzl *et al.* 1990 ([58](#_ENREF_58)) | ND | ND | ND | ND | ND | 28% | 6 |  |
|  | Henzl *et al.* 1990 ([58](#_ENREF_58)) | ND | ND | ND | ND | ND | 23% | 6 |  |
| Medical therapy after surgery | Alkatout *et al.* 2013 ([4](#_ENREF_4)) | 16% | ND | 8% | ND | 17% | ND | 12 | 16.5%  (8–23.9%) |
|  | Vercellini *et al.* 1999 ([22](#_ENREF_22)) | ND | ND | ND | ND | ND | 23.5% | 18 |  |
|  | Vlahos *et al.* 2013 ([7](#_ENREF_7)) | 20.9% | 23.9% | 17.3% | ND | ND | ND | ND |  |
|  | Vlahos *et al.* 2013 ([7](#_ENREF_7)) | 9.4% | 9.4% | 10.5% | ND | ND | ND | ND |  |

*Note:* There were no data for patients who received aromatase inhibitors, GnRH agonists plus add-back, progestins or placebo control.

GnRH, gonadotropin-releasing hormone; ND, no data; NEET, Naferelin Endometriosis European Trial.

**Supplemental Table 6.** VAS score for endometriosis-associated pain symptoms at baseline and following treatment.

| **Treatment class** | **Study** | **VAS score at baseline, cm** | **VAS score at end of treatment, cm (time)** | **VAS score after treatment cessation, cm (time)** |
| --- | --- | --- | --- | --- |
| **Pelvic pain** |  |  |  |  |
| Combined hormonal contraceptives | Zupi *et al.* 2004 ([1](#_ENREF_1)) | 6.3 | 1.9 (6 months); 0.8 (12 months) | 5.9 (6 months) |
|  | Leone Roberti Maggiore *et al.* 2014 ([10](#_ENREF_10)) | 5.6 | 3.5 (12 months) | ND |
| GnRH agonists | Zupi *et al.* 2004 ([1](#_ENREF_1)) | 6.7 | 1.3 (6 months); 0.2 (12 months) | 3.2 (6 months) |
|  | Strowitzki *et al.*  2010 ([14](#_ENREF_14)) | 5.79 | 1.19 (6 months) | ND |
| GnRH agonists plus add-back therapy | Zupi *et al.* 2004 ([1](#_ENREF_1)) | 6.9 | 1.5 (6 months); 0.3 (12 months) | 3.7 (6 months) |
| Progestins | Leone Roberti Maggiore *et al.* 2014 ([10](#_ENREF_10)) | 5.7 | 2.9 (12 months) | ND |
|  | Strowitzki *et al.* 2010 ([14](#_ENREF_14)) | 6.02 | 1.27 (6 months) | ND |
|  | Strowitzki *et al.* 2010 ([12](#_ENREF_12)) | 5.68 | 2.94 (3 months) | ND |
|  | Luisi *et al.* 2015 ([55](#_ENREF_55)) | 8.0 | 5.2 (90 days) | ND |
| Placebo control | Strowitzki *et al.* 2010 ([12](#_ENREF_12)) | 5.7 | 4.19 (3 months) | ND |
| **Dysmenrrhoea** |  |  |  |  |
| Combined hormonal contraceptives | Zupi *et al.* 2004 ([1](#_ENREF_1)) | 6.0 | 1.9 (6 months); 0.9 (12 months) | 4.9 (6 months) |
|  | Leone Roberti Maggiore *et al.* 2014 ([10](#_ENREF_10)) | 6.4 | 3.1 (12 months) | ND |
|  | Harada *et al.* 2008 ([32](#_ENREF_32)) | 5.87 | 2.76 (4 months) | ND |
| GnRH agonists | Zupi *et al.* 2004 ([1](#_ENREF_1)) | 6.1 | 0.0 (6 months); 0.0 (12 months) | 3.4 (6 months) |
| GnRH agonists plus add-back therapy | Zupi *et al.* 2004 ([1](#_ENREF_1)) | 5.8 | 0.0 (6 months); 0.0 (12 months) | 3.1 (6 months) |
| Progestins | Leone Roberti Maggiore *et al.* 2014 ([10](#_ENREF_10)) | 6.7 | ND | ND |
| Placebo control | Harada *et al.* 2008 ([32](#_ENREF_32)) | 5.58 | 4.62 (4 months) | ND |
| **Dyspareunia** |  |  |  |  |
| Combined hormonal contraceptives | Zupi *et al.* 2004 ([1](#_ENREF_1)) | 5.6 | 2.7 (6 months); 1.3 (12 months) | 3.9 (6 months) |
|  | Leone Roberti Maggiore *et al.* 2014 ([10](#_ENREF_10)) | 5.8 | 3.2 (12 months) | ND |
| GnRH agonists | Zupi *et al.* 2004 ([1](#_ENREF_1)) | 5.9 | 2.6 (6 months); 1.4 (12 months) | 2.2 (6 months) |
| GnRH agonists plus add-back therapy | Zupi *et al.* 2004 ([1](#_ENREF_1)) | 5.8 | 2.4 (6 months); 1.2 (12 months) | 2.7 (6 months) |
| Progestins | Leone Roberti Maggiore *et al.* 2014 ([10](#_ENREF_10)) | 5.4 | 2.5 (12 months) | ND |
| **Dyschezia** |  |  |  |  |
| Combined hormonal contraceptives | Leone Roberti Maggiore *et al.* 2014 ([10](#_ENREF_10)) | 5.3 | 5.1 (6 months); 4.1 (12 months) | ND |
| Progestins | Leone Roberti Maggiore *et al.* 2014 ([10](#_ENREF_10)) | 5.1 | 2.2 (6 months); 1.9 (12 months) | ND |

*Note:* Combined hormonal contraceptives comprised combined oral contraceptives, vaginal ring and contraceptive patch. There were no data for patients who received danazol, gestrinone or aromatase inhibitors.

GnRH, gonadotropin-releasing hormone; ND, no data; VAS, visual analogue scale.

**Supplemental Table 7.** Proportion of patients with no change or progression in disease score

| **Treatment class** | **Study** | **Proportion** | **Median (range)** |
| --- | --- | --- | --- |
| Danazol | Henzl et al. 1988 ([3](#_ENREF_3)) | 17% | 26.5% (6–56.1%) |
|  | Barbieri *et al.* 1982 ([8](#_ENREF_8)) | 6% |  |
|  | Naferelin Endometriosis European Trial Group 1992 ([34](#_ENREF_34)) | 11% |  |
|  | Shaw *et al. et al.* 1992 ([37](#_ENREF_37)) | 26.5% |  |
|  | Bromham *et al.* 1995 ([51](#_ENREF_51)) | 27.3% |  |
|  | Rock *et al.* 1993 ([54](#_ENREF_54)) | 56.1% |  |
|  | Bulletti *et al.* 1996 ([17](#_ENREF_17)) | 38.2% |  |
| Gestrinone | Bromham *et al.* 1995 ([51](#_ENREF_51)) | 26.7% | 26.7% |
| GnRH agonists | Reichel *et al.* 1992 ([23](#_ENREF_23)) | 4.7% | 17% (3–44.5%) |
|  | Naferelin Endometriosis European Trial Group 1992 ([34](#_ENREF_34)) | 3% |  |
|  | Shaw *et al. et al.* 1992 ([37](#_ENREF_37)) | 26.5% |  |
|  | Rock *et al.* 1993 ([54](#_ENREF_54)) | 41.3% |  |
|  | Bulletti *et al.* 1996 ([17](#_ENREF_17)) | 44.5% |  |
|  | Henzl *et al.* 1988 ([3](#_ENREF_3)) | 16% |  |
|  | Henzl *et al.* 1988 ([3](#_ENREF_3)) | 17% |  |
| Progestins | Harrison *et al.* 2000 ([18](#_ENREF_18)) | 45% | 45% |
| Placebo control | Harrison *et al.* 2000 ([18](#_ENREF_18)) | 37% | 37% |
| Medical therapy after surgery | Gerhard *et al.* 1992 ([49](#_ENREF_49)) | 45% | 45% |

*Note:* There were no data for patients who received aromatase inhibitors, combined hormonal contraceptives, GnRH agonists plus add-back therapy, GnRH antagonists or progestins.

GnRH, gonadotropin-releasing hormone.

**Supplemental Table 8.** Discontinuation rate due to adverse events or lack of efficacy

| **Treatment** | **Reference** | **Discontinuation rate** | **Treatment duration, months** | **Median (range)** |
| --- | --- | --- | --- | --- |
| Danazol | Henzl *et al.* 1988 ([3](#_ENREF_3)) | 8.8% | 6 | 9.3% (1–24.8%) |
|  | Barbieri et al. 1982 ([8](#_ENREF_8)) | 1% | 4.3 (mean) |  |
|  | Bromham *et al.* 1995 ([33](#_ENREF_33)) | 24.8% | 6 |  |
|  | NEET Group 1992 ([34](#_ENREF_34)) | 4% | 6 |  |
|  | Rolland *et al.* 1990 ([35](#_ENREF_35)) | 3% | 6 |  |
|  | Shaw *et al.* 1992 ([37](#_ENREF_37)) | 9.7% | 6 |  |
|  | Bromham *et al.* 1995 ([51](#_ENREF_51)) | 12.4% | 6 |  |
|  | Rock *et al.* 1993 ([54](#_ENREF_54)) | 12.1% | 6 |  |
| Gestrinone | Bromham *et al.* 1995 ([33](#_ENREF_33)) | 20.5% | 6 | 16.0% (11.4–20.5%) |
|  | Bromham *et al.* 1995 ([51](#_ENREF_51)) | 11.4% | 6 |  |
| Combined hormonal contraceptives | Vercellini *et al.* 2010 ([6](#_ENREF_6)) | 13.0% (vaginal ring); 23.8% (contraceptive patch) | 12 | 8.3% (4.9–24.4%) |
|  | Vlahos *et al.* 2013 ([7](#_ENREF_7)) | 8.3% (cyclic COC); 12.9% (continuous COC) | 12 |  |
|  | Maggiore *et al.* 2014 ([10](#_ENREF_10)) | 14.5% (vaginal ring) | 12 |  |
|  | Seracchioli *et al.* 2010 ([29](#_ENREF_29)) | 4.9% (cyclic COC); 5.1% (continuous COC) | 24 |  |
|  | Seracchioli *et al.* 2010 ([30](#_ENREF_30)) | 7.8% (cyclic COC); 5.8% (continuous COC) | 24 |  |
|  | Harada *et al.* 2008 ([32](#_ENREF_32)) | 7.8% | 4 |  |
|  | Morotti *et al.* 2014 ([40](#_ENREF_40)) | 24.4% | 6 |  |
| GnRH agonists | Henzl *et al.* 1988 ([3](#_ENREF_3)) | 8.9% (800 µg nafarelin);  0.0% (400 µg nafarelin) | 6 | 4.9% (0–25%) |
|  | Bergqvist *et al.* 2000 ([5](#_ENREF_5)) | 6.9% (goserelin); 7.4% (nafarelin) | 6 |  |
|  | Strowitzki *et al.* 2010 ([14](#_ENREF_14)) | 3.9% | 6 |  |
|  | Reichel *et al.* 1992 ([23](#_ENREF_23)) | 5.5% | 6 |  |
|  | NEET Group, 1992 ([34](#_ENREF_34)) | 4.9% | 6 |  |
|  | Rolland *et al.* 1990 ([35](#_ENREF_35)) | 5.5% | 6 |  |
|  | Agarwal *et al.* 1997 ([36](#_ENREF_36)) | 2.9% (nafarelin); 5.1% (leuprolide) | 6 |  |
|  | Shaw *et al.* 1992 ([37](#_ENREF_37)) | 1.0% | 6 |  |
|  | Ling 1999 ([42](#_ENREF_42)) | 0% | 6 |  |
|  | Crosignani *et al.* 2006 ([45](#_ENREF_45)) | 1.4% | 6 |  |
|  | Schlaff *et al.* 2006 ([46](#_ENREF_46)) | 6.5% | 6 |  |
|  | Gerhard *et al.* 1992 ([49](#_ENREF_49)) | 3.8% | 6 |  |
|  | Hornstein *et al.* 1997 ([52](#_ENREF_52)) | 25% | 6 |  |
|  | Rock *et al.* 1993 ([54](#_ENREF_54)) | 2.9% | 6 |  |
| GnRH agonists plus add-back therapy | Tsai *et al.*  2016 ([57](#_ENREF_57)) | 9.6% (1 mg estradiol + 2.5 mg medroxyprogesterone); 14.5% (2 mg estradiol + 5 mg medroxyprogesterone) | 5 | 12.1%  (9.6–14.5%) |
| GnRH antagonists | Diamond *et al.* 2014 ([19](#_ENREF_19)) | 5.8% (150 mg elagolix);  6.9% (250 mg elagolix) | 6 | 5.8% (4.8–8.3%) |
|  | Carr *et al.* 2013 ([20](#_ENREF_20)) | 4.8% | 6 |  |
|  | Carr *et al.* 2014 ([21](#_ENREF_21)) | 4.8%; 8.3% | 6 |  |
| Progestins | Maggiore *et al.* 2014 ([10](#_ENREF_10)) | 10.0% | 12 | 7.0% (0.0–30.0%) |
|  | Strowitzki *et al.* 2010 ([12](#_ENREF_12)) | 2.0% | 3 |  |
|  | Strowitzki *et al.* 2010 ([14](#_ENREF_14)) | 5.0% | 6 |  |
|  | Harrison *et al.* 2000 ([18](#_ENREF_18)) | 0.0% | 3 |  |
|  | Carr *et al.* 2014 ([21](#_ENREF_21)) | 16.7% | 6 |  |
|  | Momoeda *et al.* 2009 ([31](#_ENREF_31)) | 7.4% | 12 |  |
|  | Morotti *et al.* 2014 ([40](#_ENREF_40)) | 11.3% | 6 |  |
|  | Crosignani *et al.* 2006 ([45](#_ENREF_45)) | 2.0% | 6 |  |
|  | Schlaff *et al.* 2006 ([46](#_ENREF_46)) | 6.6% | 6 |  |
|  | Luisi *et al.*  2015 ([55](#_ENREF_55)) | 4% | 3 |  |
|  | Vercellini *et al.*  2016 ([59](#_ENREF_59)) | 24% (norethindrone acetate);  30% (dienogest) | 6 |  |
| Placebo control | Strowitzki *et al.* 2010 ([12](#_ENREF_12)) | 1.0% | 3 | 1.4% (0.0–71.0%) |
|  | Hornstein *et al.* 1997 ([52](#_ENREF_52)) | 47% | 6 |  |
|  | Harrison *et al.* 2000 ([18](#_ENREF_18)) | 0.0% | 3 |  |
|  | Diamond *et al.* 2014 ([19](#_ENREF_19)) | 5.8% | 3 |  |
|  | Carr *et al.* 2013 ([20](#_ENREF_20)) | 1.4% | 2 |  |
|  | Harada *et al.* 2008 ([32](#_ENREF_32)) | 6.1% | 4 |  |
|  | Roghaei *et al.* 2010 ([16](#_ENREF_16)) | 71.0% | 6 |  |
|  | Ling 1999 ([42](#_ENREF_42)) | 0.0% | 3 |  |
|  | Ling 1999 ([42](#_ENREF_42)) | 0.0% | 6 |  |
| Medical therapy after surgery | Gerhard *et al.* 1992 ([49](#_ENREF_49)) | 3.8% | 6 | 5.5%  (3.8–71.0%) |
|  | Seracchioli *et al.* 2010 ([29](#_ENREF_29)) | 4.9%, 5.1% | 24 |  |
|  | Seracchioli *et al.* 2010 ([30](#_ENREF_30)) | 7.8%, 5.8% | 24 |  |
|  | Roghaei *et al.* 2010 ([16](#_ENREF_16)) | 71.0% | 6 |  |

*Note:* Combined hormonal contraceptives comprised combined oral contraceptives, vaginal ring and patch. There were no data for patients who received GnRH agonists plus add-back therapy.

*Includes patients who received placebo control or dietary therapy.

COC, combined oral contraceptives; GnRH, gonadotropin-releasing hormone.

**REFERENCES**

1. Zupi E, Marconi D, Sbracia M, Zullo F, De Vivo B, Exacustos C, et al. Add-back therapy in the treatment of endometriosis-associated pain. Fertil Steril 2004;82:1303–8.

2. Donnez J, Nisolle-Pochet M, Clerckx-Braun F, Sandow J, Casanas-Roux F. Administration of nasal buserelin as compared with subcutaneous buserelin implant for endometriosis. Fertil Steril 1989;52:27–30.

3. Henzl MR, Corson SL, Moghissi K, Buttram VC, Berqvist C, Jacobson J. Administration of nasal nafarelin as compared with oral danazol for endometriosis. A multicenter double-blind comparative clinical trial. N Engl J Med 1988;318:485–9.

4. Alkatout I, Mettler L, Beteta C, Hedderich J, Jonat W, Schollmeyer T, et al. Combined surgical and hormone therapy for endometriosis is the most effective treatment: prospective, randomized, controlled trial. J Minim Invasive Gynecol 2013;20:473–81.

5. Bergqvist A, Petersson F, Bergquist C, Elfgren K, Hahn L, Lalos O, et al. A comparative study of the acceptability and effect of goserelin and nafarelin on endometriosis. Gynecol Endocrinol 2000;14:425–32.

6. Vercellini P, Barbara G, Somigliana E, Bianchi S, Abbiati A, Fedele L. Comparison of contraceptive ring and patch for the treatment of symptomatic endometriosis. Fertil Steril 2010;93:2150–61.

7. Vlahos N, Vlachos A, Triantafyllidou O, Vitoratos N, Creatsas G. Continuous versus cyclic use of oral contraceptives after surgery for symptomatic endometriosis: a prospective cohort study. Fertil Steril 2013;100:1337–42.

8. Barbieri RL, Evans S, Kistner RW. Danazol in the treatment of endometriosis: analysis of 100 cases with a 4-year follow-up. Fertil Steril 1982;37:737–46.

9. Wheeler JM, Knittle JD, Miller JD. Depot leuprolide versus danazol in treatment of women with symptomatic endometriosis. I. Efficacy results. Am J Obstet Gynecol 1992;167:1367–71.

10. Leone Roberti Maggiore U, Remorgida V, Scala C, Tafi E, Venturini PL, Ferrero S. Desogestrel-only contraceptive pill versus sequential contraceptive vaginal ring in the treatment of rectovaginal endometriosis infiltrating the rectum: a prospective open-label comparative study. Acta Obstet Gynecol Scand 2014;93:239–47.

11. Strowitzki T, Marr J, Gerlinger C, Faustmann T, Seitz C. Detailed analysis of a randomized, multicenter, comparative trial of dienogest versus leuprolide acetate in endometriosis. Int J Gynaecol Obstet 2012;117:228–33.

12. Strowitzki T, Faustmann T, Gerlinger C, Seitz C. Dienogest in the treatment of endometriosis-associated pelvic pain: a 12-week, randomized, double-blind, placebo-controlled study. Eur J Obstet Gynecol Reprod Biol 2010;151:193–8.

13. Harada T, Momoeda M, Taketani Y, Aso T, Fukunaga M, Hagino H, et al. Dienogest is as effective as intranasal buserelin acetate for the relief of pain symptoms associated with endometriosis − a randomized, double-blind, multicenter, controlled trial. Fertil Steril 2009;91:675–81.

14. Strowitzki T, Marr J, Gerlinger C, Faustmann T, Seitz C. Dienogest is as effective as leuprolide acetate in treating the painful symptoms of endometriosis: a 24-week, randomized, multicentre, open-label trial. Hum Reprod 2010;25:633–41.

15. Yang XH, Ji F, AiLi A, TuerXun H, He Y, Ding Y. Effects of laparoscopic ovarian endometriosis cystectomy combined with postoperative GnRH-a therapy on ovarian reserve, pregnancy, and outcome recurrence. Clin Exp Obstet Gynecol 2014;41:272–5.

16. Roghaei MA, Tehrany HG, Taherian A, Koleini N. Effects of letrozole compared with danazol on patients with confirmed endometriosis: a randomized clinical trial. Int J Fertil Menopausal Stud 2010;4:67–72.

17. Bulletti C, Flamigni C, Polli V, Giacomucci E, Albonetti A, Negrini V, et al. The efficacy of drugs in the management of endometriosis. J Am Assoc Gynecol Laparosc 1996;3:495–501.

18. Harrison RF, Barry-Kinsella C. Efficacy of medroxyprogesterone treatment in infertile women with endometriosis: a prospective, randomized, placebo-controlled study. Fertil Steril 2000;74:24–30.

19. Diamond MP, Carr B, Dmowski WP, Koltun W, O'Brien C, Jiang P, et al. Elagolix treatment for endometriosis-associated pain: results from a phase 2, randomized, double-blind, placebo-controlled study. Reprod Sci 2014;21:363–71.

20. Carr B, Giudice L, Dmowski WP, O'Brien C, Jiang P, Burke J, et al. Elagolix, an oral GnRH antagonist for endometriosis associated pain: a randomized controlled study. J Endometriosis 2013;5:105–15.

21. Carr B, Dmowski WP, O'Brien C, Jiang P, Burke J, Jimenez R, et al. Elagolix, an oral GnRH antagonist, versus subcutaneous depot medroxyprogesterone acetate for the treatment of endometriosis: effects on bone mineral density. Reprod Sci 2014;21:1341–51.

22. Vercellini P, Crosignani PG, Fadini R, Radici E, Belloni C, Sismondi P. A gonadotrophin-releasing hormone agonist compared with expectant management after conservative surgery for symptomatic endometriosis. Br J Obstet Gynaecol 1999;106:672–7.

23. Reichel RP, Schweppe KW. Goserelin (Zoladex) depot in the treatment of endometriosis. Zoladex Endometriosis Study Group. Fertil Steril 1992;57:1197–202.

24. Moghissi KS, Schlaff WD, Olive DL, Skinner MA, Yin H. Goserelin acetate (Zoladex) with or without hormone replacement therapy for the treatment of endometriosis. Fertil Steril 1998;69:1056–62.

25. Miller JD, Shaw RW, Casper RFJ, Rock JA, Thomas EJ, Dmowski WP, et al. Historical prospective cohort study of the recurrence of pain after discontinuation of treatment with danazol or a gonadotropin-releasing hormone agonist. Fertil Steril 1998;70:293–6.

26. Sesti F, Pietropolli A, Capozzolo T, Broccoli P, Pierangeli S, Bollea MR, et al. Hormonal suppression treatment or dietary therapy versus placebo in the control of painful symptoms after conservative surgery for endometriosis stage III−IV. A randomized comparative trial. Fertil Steril 2007;88:1541–7.

27. Szendei G, Hernadi Z, Devenyi N, Csapo Z. Is there any correlation between stages of endometriosis and severity of chronic pelvic pain? Possibilities of treatment. Gynecol Endocrinol 2005;21:93–100.

28. Hornstein MD, Surrey ES, Weisberg GW, Casino LA. Leuprolide acetate depot and hormonal add-back in endometriosis: a 12- month study. Obstet Gynecol 1998;91:16–24.

29. Seracchioli R, Mabrouk M, Frasca C, Manuzzi L, Montanari G, Keramyda A, et al. Long-term cyclic and continuous oral contraceptive therapy and endometrioma recurrence: a randomized controlled trial. Fertil Steril 2010;93:52–6.

30. Seracchioli R, Mabrouk M, Frasca C, Manuzzi L, Savelli L, Venturoli S. Long-term oral contraceptive pills and postoperative pain management after laparoscopic excision of ovarian endometrioma: a randomized controlled trial. Fertil Steril 2010;94:464–71.

31. Momoeda M, Harada T, Terakawa N, Aso T, Fukunaga M, Hagino H, et al. Long-term use of dienogest for the treatment of endometriosis. J Obstet Gynaecol Res 2009;35:1069–76.

32. Harada T, Momoeda M, Taketani Y, Hoshiai H, Terakawa N. Low-dose oral contraceptive pill for dysmenorrhea associated with endometriosis: a placebo-controlled, double-blind, randomized trial. Fertil Steril 2008;90:1583–8.

33. Bromham DR, Booker MW, Rose GL, Wardle PG, Newton JR. A multicentre comparative study of gestrinone and danazol in the treatment of endometriosis. J Obstet Gynaecol 1995;15:188–94.

34. Nafarelin European Endometriosis Trial Group. Nafarelin for endometriosis: a large-scale, danazol-controlled trial of efficacy and safety, with 1-year follow-up. The Nafarelin European Endometriosis Trial Group (NEET). Fertil Steril 1992;57:514–22.

35. Rolland R, van der Heijden PF. Nafarelin versus danazol in the treatment of endometriosis. Am J Obstet Gynecol 1990;162:586–8.

36. Agarwal SK, Hamrang C, Henzl MR, Judd HL. Nafarelin vs. leuprolide acetate depot for endometriosis: changes in bone mineral density and vasomotor symptoms. J Reprod Med 1997;42:413–23.

37. Shaw RW. An open randomized comparative study of the effect of goserelin depot and danazol in the treatment of endometriosis. Zoladex Endometriosis Study Team. Fertil Steril 1992;58:265–72.

38. Adamson GD, Kwei L, Edgren RA. Pain of endometriosis: effects of nafarelin and danazol therapy. Int J Fertil Menopausal Stud 1994;39:215–7.

39. Lee DY, Bae DS, Yoon BK, Choi D. Post-operative cyclic oral contraceptive use after gonadotrophin-releasing hormone agonist treatment effectively prevents endometrioma recurrence. Hum Reprod 2010;25:3050–4.

40. Morotti M, Remorgida V, Venturini PL, Ferrero S. Progestogen-only contraceptive pill compared with combined oral contraceptive in the treatment of pain symptoms caused by endometriosis in patients with migraine without aura. Eur J Obstet Gynecol Reprod Biol 2014;179:63–8.

41. Hornstein MD, Yuzpe AA, Burry KA, Heinrichs LR, Buttram Jr VL, Orwoll ES. Prospective randomized double-blind trial of 3 versus 6 months of nafarelin therapy for endometriosis associated pelvic pain. Fertil Steril 1995;63:955–62.

42. Ling FW. Randomized controlled trial of depot leuprolide in patients with chronic pelvic pain and clinically suspected endometriosis. Pelvic Pain Study Group. Obstet Gynecol 1999;93:51–8.

43. Petraglia F, Hornung D, Seitz C, Faustmann T, Gerlinger C, Luisi S, et al. Reduced pelvic pain in women with endometriosis: efficacy of long-term dienogest treatment. Arch Gynecol Obstet 2012;285:167–73.

44. Doberl A, Bergqvist A, Jeppsson S. Regression of endometriosis following shorter treatment with, or lower dose of danazol. Comparison of pre- and post-treatment laparoscopic findings in the Scandinavian multi-center study. Acta Obstet Gynecol Scand 1984;63 (Suppl 123):51–8.

45. Crosignani PG, Luciano A, Ray A, Bergqvist A. Subcutaneous depot medroxyprogesterone acetate versus leuprolide acetate in the treatment of endometriosis-associated pain. Hum Reprod 2006;21:248–56.

46. Schlaff WD, Carson SA, Luciano A, Ross D, Bergqvist A. Subcutaneous injection of depot medroxyprogesterone acetate compared with leuprolide acetate in the treatment of endometriosis-associated pain. Fertil Steril 2006;85:314–25.

47. Vercellini P, Somigliana E, Consonni D, Frattaruolo MP, De Giorgi O, Fedele L. Surgical versus medical treatment for endometriosis-associated severe deep dyspareunia: I. Effect on pain during intercourse and patient satisfaction. Hum Reprod 2012;27:3450–9.

48. Gokmen O, Ugur M. Treatment of endometriosis with Gn-Rh agonist triptorelin: a multicenter study. Turk J Med Sci 1996;26:261–6.

49. Gerhard I, Schindler AE, Buhler K, Winkler U, Meinen K, Mancarella D, et al. Treatment of endometriosis with leuprorelin acetate depot: a German multicentre study. Clin Ther 1992;14 (Suppl A):3–16.

50. Halbe HW, Nakamura MS, Da Silveira GPG, Carvalho WPC. Updating the clinical experience in endometriosis − the Brazilian perspective. Br J Obstet Gynaecol 1995;102:17–21.

51. Bromham DR, Booker MW, Rose GL, Wardle PG, Newton JR. Updating the clinical experience in endometriosis − the European perspective. Br J Obstet Gynaecol 1995;102 (Suppl 12):12–6.

52. Hornstein MD, Hemmings R, Yuzpe AA, LeRoy Heinrichs W. Use of nafarelin versus placebo after reductive laparoscopic surgery for endometriosis. Fertil Steril 1997;68:860–4.

53. Kaser DJ, Missmer SA, Berry KF, Laufer MR. Use of norethindrone acetate alone for postoperative suppression of endometriosis symptoms. J Pediatr Adolesc Gynecol 2012;25:105–8.

54. Rock JA, Truglia JA, Caplan RJ. Zoladex (goserelin acetate implant) in the treatment of endometriosis: a randomized comparison with danazol. The Zoladex Endometriosis Study Group. Obstet Gynecol 1993;82:198–205.

55. Henzl MR, Kwei L. Efficacy and safety of nafarelin in the treatment of endometriosis. Am J Obstet Gynecol 1990;162:570–74.

1. Zupi E, Marconi D, Sbracia M, Zullo F, De Vivo B, Exacustos C, et al. Add-back therapy in the treatment of endometriosis-associated pain. Fertil Steril 2004;82:1303−8.

2. Donnez J, Nisolle-Pochet M, Clerckx-Braun F, Sandow J, Casanas-Roux F. Administration of nasal buserelin as compared with subcutaneous buserelin implant for endometriosis. Fertil Steril 1989;52:27−30.

3. Henzl MR, Corson SL, Moghissi K, Buttram VC, Berqvist C, Jacobson J. Administration of nasal nafarelin as compared with oral danazol for endometriosis. A multicenter double-blind comparative clinical trial. N Engl J Med 1988;318:485−9.

4. Alkatout I, Mettler L, Beteta C, Hedderich J, Jonat W, Schollmeyer T, et al. Combined surgical and hormone therapy for endometriosis is the most effective treatment: prospective, randomized, controlled trial. J Minim Invasive Gynecol 2013;20:473−81.

5. Bergqvist A, Petersson F, Bergquist C, Elfgren K, Hahn L, Lalos O, et al. A comparative study of the acceptability and effect of goserelin and nafarelin on endometriosis. Gynecol Endocrinol 2000;14:425−32.

6. Vercellini P, Barbara G, Somigliana E, Bianchi S, Abbiati A, Fedele L. Comparison of contraceptive ring and patch for the treatment of symptomatic endometriosis. Fertil Steril 2010;93:2150−61.

7. Vlahos N, Vlachos A, Triantafyllidou O, Vitoratos N, Creatsas G. Continuous versus cyclic use of oral contraceptives after surgery for symptomatic endometriosis: a prospective cohort study. Fertil Steril 2013;100:1337−42.

8. Barbieri RL, Evans S, Kistner RW. Danazol in the treatment of endometriosis: analysis of 100 cases with a 4-year follow-up. Fertil Steril 1982;37:737−46.

9. Wheeler JM, Knittle JD, Miller JD. Depot leuprolide versus danazol in treatment of women with symptomatic endometriosis. I. Efficacy results. Am J Obstet Gynecol 1992;167:1367−71.

10. Leone Roberti Maggiore U, Remorgida V, Scala C, Tafi E, Venturini PL, Ferrero S. Desogestrel-only contraceptive pill versus sequential contraceptive vaginal ring in the treatment of rectovaginal endometriosis infiltrating the rectum: a prospective open-label comparative study. Acta Obstet Gynecol Scand 2014;93:239−47.

11. Strowitzki T, Marr J, Gerlinger C, Faustmann T, Seitz C. Detailed analysis of a randomized, multicenter, comparative trial of dienogest versus leuprolide acetate in endometriosis. Int J Gynaecol Obstet 2012;117:228−33.

12. Strowitzki T, Faustmann T, Gerlinger C, Seitz C. Dienogest in the treatment of endometriosis-associated pelvic pain: a 12-week, randomized, double-blind, placebo-controlled study. Eur J Obstet Gynecol Reprod Biol 2010;151:193−8.

13. Harada T, Momoeda M, Taketani Y, Aso T, Fukunaga M, Hagino H, et al. Dienogest is as effective as intranasal buserelin acetate for the relief of pain symptoms associated with endometriosis − a randomized, double-blind, multicenter, controlled trial. Fertil Steril 2009;91:675−81.

14. Strowitzki T, Marr J, Gerlinger C, Faustmann T, Seitz C. Dienogest is as effective as leuprolide acetate in treating the painful symptoms of endometriosis: a 24-week, randomized, multicentre, open-label trial. Hum Reprod 2010;25:633−41.

15. Yang XH, Ji F, AiLi A, TuerXun H, He Y, Ding Y. Effects of laparoscopic ovarian endometriosis cystectomy combined with postoperative GnRH-a therapy on ovarian reserve, pregnancy, and outcome recurrence. Clin Exp Obstet Gynecol 2014;41:272−5.

16. Roghaei MA, Tehrany HG, Taherian A, Koleini N. Effects of letrozole compared with danazol on patients with confirmed endometriosis: a randomized clinical trial. Int J Fertil Menopausal Stud 2010;4:67−72.

17. Bulletti C, Flamigni C, Polli V, Giacomucci E, Albonetti A, Negrini V, et al. The efficacy of drugs in the management of endometriosis. J Am Assoc Gynecol Laparosc 1996;3:495−501.

18. Harrison RF, Barry-Kinsella C. Efficacy of medroxyprogesterone treatment in infertile women with endometriosis: a prospective, randomized, placebo-controlled study. Fertil Steril 2000;74:24−30.

19. Diamond MP, Carr B, Dmowski WP, Koltun W, O'Brien C, Jiang P, et al. Elagolix treatment for endometriosis-associated pain: results from a phase 2, randomized, double-blind, placebo-controlled study. Reprod Sci 2014;21:363−71.

20. Carr B, Giudice L, Dmowski WP, O'Brien C, Jiang P, Burke J, et al. Elagolix, an oral GnRH antagonist for endometriosis associated pain: a randomized controlled study. J Endometriosis 2013;5:105−15.

21. Carr B, Dmowski WP, O'Brien C, Jiang P, Burke J, Jimenez R, et al. Elagolix, an oral GnRH antagonist, versus subcutaneous depot medroxyprogesterone acetate for the treatment of endometriosis: effects on bone mineral density. Reprod Sci 2014;21:1341−51.

22. Vercellini P, Crosignani PG, Fadini R, Radici E, Belloni C, Sismondi P. A gonadotrophin-releasing hormone agonist compared with expectant management after conservative surgery for symptomatic endometriosis. Br J Obstet Gynaecol 1999;106:672−7.

23. Reichel RP, Schweppe KW. Goserelin (Zoladex) depot in the treatment of endometriosis. Zoladex Endometriosis Study Group. Fertil Steril 1992;57:1197−202.

24. Moghissi KS, Schlaff WD, Olive DL, Skinner MA, Yin H. Goserelin acetate (Zoladex) with or without hormone replacement therapy for the treatment of endometriosis. Fertil Steril 1998;69:1056−62.

25. Miller JD, Shaw RW, Casper RFJ, Rock JA, Thomas EJ, Dmowski WP, et al. Historical prospective cohort study of the recurrence of pain after discontinuation of treatment with danazol or a gonadotropin-releasing hormone agonist. Fertil Steril 1998;70:293−6.

26. Sesti F, Pietropolli A, Capozzolo T, Broccoli P, Pierangeli S, Bollea MR, et al. Hormonal suppression treatment or dietary therapy versus placebo in the control of painful symptoms after conservative surgery for endometriosis stage III−IV. A randomized comparative trial. Fertil Steril 2007;88:1541−7.

27. Szendei G, Hernadi Z, Devenyi N, Csapo Z. Is there any correlation between stages of endometriosis and severity of chronic pelvic pain? Possibilities of treatment. Gynecol Endocrinol 2005;21:93−100.

28. Hornstein MD, Surrey ES, Weisberg GW, Casino LA. Leuprolide acetate depot and hormonal add-back in endometriosis: a 12- month study. Obstet Gynecol 1998;91:16−24.

29. Seracchioli R, Mabrouk M, Frasca C, Manuzzi L, Montanari G, Keramyda A, et al. Long-term cyclic and continuous oral contraceptive therapy and endometrioma recurrence: a randomized controlled trial. Fertil Steril 2010;93:52−6.

30. Seracchioli R, Mabrouk M, Frasca C, Manuzzi L, Savelli L, Venturoli S. Long-term oral contraceptive pills and postoperative pain management after laparoscopic excision of ovarian endometrioma: a randomized controlled trial. Fertil Steril 2010;94:464−71.

31. Momoeda M, Harada T, Terakawa N, Aso T, Fukunaga M, Hagino H, et al. Long-term use of dienogest for the treatment of endometriosis. J Obstet Gynaecol Res 2009;35:1069−76.

32. Harada T, Momoeda M, Taketani Y, Hoshiai H, Terakawa N. Low-dose oral contraceptive pill for dysmenorrhea associated with endometriosis: a placebo-controlled, double-blind, randomized trial. Fertil Steril 2008;90:1583−8.

33. Bromham DR, Booker MW, Rose GL, Wardle PG, Newton JR. A multicentre comparative study of gestrinone and danazol in the treatment of endometriosis. J Obstet Gynaecol 1995;15:188−94.

34. Nafarelin European Endometriosis Trial Group. Nafarelin for endometriosis: a large-scale, danazol-controlled trial of efficacy and safety, with 1-year follow-up. The Nafarelin European Endometriosis Trial Group (NEET). Fertil Steril 1992;57:514−22.

35. Rolland R, van der Heijden PF. Nafarelin versus danazol in the treatment of endometriosis. Am J Obstet Gynecol 1990;162:586−8.

36. Agarwal SK, Hamrang C, Henzl MR, Judd HL. Nafarelin vs. leuprolide acetate depot for endometriosis: changes in bone mineral density and vasomotor symptoms. J Reprod Med 1997;42:413−23.

37. Shaw RW. An open randomized comparative study of the effect of goserelin depot and danazol in the treatment of endometriosis. Zoladex Endometriosis Study Team. Fertil Steril 1992;58:265−72.

38. Adamson GD, Kwei L, Edgren RA. Pain of endometriosis: effects of nafarelin and danazol therapy. Int J Fertil Menopausal Stud 1994;39:215−7.

39. Lee DY, Bae DS, Yoon BK, Choi D. Post-operative cyclic oral contraceptive use after gonadotrophin-releasing hormone agonist treatment effectively prevents endometrioma recurrence. Hum Reprod 2010;25:3050−4.

40. Morotti M, Remorgida V, Venturini PL, Ferrero S. Progestogen-only contraceptive pill compared with combined oral contraceptive in the treatment of pain symptoms caused by endometriosis in patients with migraine without aura. Eur J Obstet Gynecol Reprod Biol 2014;179:63−8.

41. Hornstein MD, Yuzpe AA, Burry KA, Heinrichs LR, Buttram Jr VL, Orwoll ES. Prospective randomized double-blind trial of 3 versus 6 months of nafarelin therapy for endometriosis associated pelvic pain. Fertil Steril 1995;63:955−62.

42. Ling FW. Randomized controlled trial of depot leuprolide in patients with chronic pelvic pain and clinically suspected endometriosis. Pelvic Pain Study Group. Obstet Gynecol 1999;93:51−8.

43. Petraglia F, Hornung D, Seitz C, Faustmann T, Gerlinger C, Luisi S, et al. Reduced pelvic pain in women with endometriosis: efficacy of long-term dienogest treatment. Arch Gynecol Obstet 2012;285:167−73.

44. Doberl A, Bergqvist A, Jeppsson S. Regression of endometriosis following shorter treatment with, or lower dose of danazol. Comparison of pre- and post-treatment laparoscopic findings in the Scandinavian multi-center study. Acta Obstet Gynecol Scand 1984;63 (Suppl 123):51−8.

45. Crosignani PG, Luciano A, Ray A, Bergqvist A. Subcutaneous depot medroxyprogesterone acetate versus leuprolide acetate in the treatment of endometriosis-associated pain. Hum Reprod 2006;21:248−56.

46. Schlaff WD, Carson SA, Luciano A, Ross D, Bergqvist A. Subcutaneous injection of depot medroxyprogesterone acetate compared with leuprolide acetate in the treatment of endometriosis-associated pain. Fertil Steril 2006;85:314−25.

47. Vercellini P, Somigliana E, Consonni D, Frattaruolo MP, De Giorgi O, Fedele L. Surgical versus medical treatment for endometriosis-associated severe deep dyspareunia: I. Effect on pain during intercourse and patient satisfaction. Hum Reprod 2012;27:3450−9.

48. Gokmen O, Ugur M. Treatment of endometriosis with Gn-Rh agonist triptorelin: a multicenter study. Turk J Med Sci 1996;26:261−6.

49. Gerhard I, Schindler AE, Buhler K, Winkler U, Meinen K, Mancarella D, et al. Treatment of endometriosis with leuprorelin acetate depot: a German multicentre study. Clin Ther 1992;14 (Suppl A):3−16.

50. Halbe HW, Nakamura MS, Da Silveira GPG, Carvalho WPC. Updating the clinical experience in endometriosis − the Brazilian perspective. Br J Obstet Gynaecol 1995;102:17−21.

51. Bromham DR, Booker MW, Rose GL, Wardle PG, Newton JR. Updating the clinical experience in endometriosis − the European perspective. Br J Obstet Gynaecol 1995;102 (Suppl 12):12−6.

52. Hornstein MD, Hemmings R, Yuzpe AA, LeRoy Heinrichs W. Use of nafarelin versus placebo after reductive laparoscopic surgery for endometriosis. Fertil Steril 1997;68:860−4.

53. Kaser DJ, Missmer SA, Berry KF, Laufer MR. Use of norethindrone acetate alone for postoperative suppression of endometriosis symptoms. J Pediatr Adolesc Gynecol 2012;25:105−8.

54. Rock JA, Truglia JA, Caplan RJ. Zoladex (goserelin acetate implant) in the treatment of endometriosis: a randomized comparison with danazol. The Zoladex Endometriosis Study Group. Obstet Gynecol 1993;82:198−205.

55. Luisi S, Parazzini F, Angioni S, Arena S, Berretta P, Candiani M, et al. Dienogest treatment improves quality of life in women with endometriosis. Journal of Endometriosis and Pelvic Pain Disorders 2015;7:124−28.

56. Carbonell JL, Riveron AM, Leonard Y, Gonzalez J, Heredia B, Sanchez C. Mifepristone 2.5, 5, 10 mg versus placebo in the treatment of endometriosis. Journal of Reproductive Health and Medicine 2016;2:17−25.

57. Tsai HW, Wang PH, Huang BS, Twu NF, Yen MS, Chen YJ. Low-dose add-back therapy during postoperative GnRH agonist treatment. Taiwan J Obstet Gynecol 2016;55:55-9.

58. Henzl MR, Kwei L. Efficacy and safety of nafarelin in the treatment of endometriosis. Am J Obstet Gynecol 1990;162:570−74.

59. Vercellini P, Bracco B, Mosconi P, Roberto A, Alberico D, Dhouha D, et al. Norethindrone acetate or dienogest for the treatment of symptomatic endometriosis: a before and after study. Fertil Steril 2016;105:734-43 e3.
